# Bioprospection of Tenellins Produced by the Entomopathogenic Fungus *Beauveria neobassiana*

**DOI:** 10.3390/jof10010069

**Published:** 2024-01-15

**Authors:** Rita Toshe, Esteban Charria-Girón, Artit Khonsanit, Janet Jennifer Luangsa-ard, Syeda Javariya Khalid, Hedda Schrey, Sherif S. Ebada, Marc Stadler

**Affiliations:** 1 Department of Microbial Drugs, Helmholtz Centre for Infection Research GmbH (HZI), Inhoffenstraße 7, 38124 Braunschweig, Germany; rita.toshe@helmholtz-hzi.de (R.T.); esteban.charriagiron@helmholtz-hzi.de (E.C.-G.); syeda.khalid@helmholtz-hzi.de (S.J.K.); hedda.schrey@helmholtz-hzi.de (H.S.); 2 Institute of Microbiology, Technische Universität Braunschweig, Spielmannstraße 7, 38106 Braunschweig, Germany; 3 National Center for Genetic Engineering and Biotechnology (BIOTEC), National Science and Technology Development Agency (NSTDA), 113 Thailand Science Park, Phahonyothin Rd., Khlong Nueng, Khlong Luang, Pathum Thani 12120, Thailand; artit.kho@biotec.or.th (A.K.); jajen@biotec.or.th (J.J.L.-a.); 4 Department of Pharmacognosy, Faculty of Pharmacy, Ain Shams University, Cairo 11566, Egypt

**Keywords:** Cordycipitaceae, insect fungi, biofilm inhibition, antiproliferative, SAR, PKS-NRPS

## Abstract

Fungi are known as prolific producers of bioactive secondary metabolites with applications across various fields, including infectious diseases, as well as in biological control. However, some of the well-known species are still underexplored. Our current study evaluated the production of secondary metabolites by the entomopathogenic fungus *Beauveria neobassiana* from Thailand. The fermentation of this fungus in a liquid medium, followed by preparative high-performance liquid chromatography (HPLC) purification, resulted in the isolation of a new tenellin congener, namely pretenellin C (**1**), together with five known derivatives (**2**–**6**). Their chemical structures were elucidated by 1D and 2D nuclear magnetic resonance (NMR) spectroscopy in combination with high-resolution electrospray ionization mass spectrometry (HR-ESI-MS). We evaluated the antimicrobial and cytotoxic activities from all isolated compounds, as well as their inhibitory properties on biofilm formation by *Staphylococcus aureus*. Generally, tenellins displayed varying antibiofilm and cytotoxic effects, allowing us to propose preliminary structure-activity relationships (SARs). Among the tested compounds, prototenellin D (**2**) exhibited the most prominent antibiofilm activity, while its 2-pyridone congener, tenellin (**4**), demonstrated potent cytotoxic activity against all tested cell lines. Given the fact that the biological activities of the tenellins have so far been neglected in the past, our study could provide a good starting point to establish more concise structure-activity relationships in the near future.

## 1. Introduction

Fungi, renowned for their valuable contributions across a range of different fields, are also recognized for being creative secondary metabolite producers. These compounds have significantly benefited society, particularly in antibiotic development, as exemplified by the pleuromutilins, one of the most recent antibiotics class introduced to the market [1]. However, the value of these organisms extends beyond the control of infectious diseases, as fungi are instrumental in a wealth of applications, such as in agriculture, where they serve as biocontrol agents [2]. The interactions within fungal ecosystems have been leveraged to create environmentally friendly alternatives for pest control, as in the case of entomopathogenic fungi, which act against specific pests while sparing other organisms from harm [3].

Species belonging to the genus *Beauveria* (Cordycipitaceae, Ascomycota) are widely employed in agriculture as biocontrol agents [4]. Recent taxonomic revisions of this genus, facilitated by a combination of whole-genome sequencing, morphometric analysis, and chemical studies, have aided in the delimitation of the *Beauveria asiatica* and *Beauveria bassiana* (*sensu lato)* species complexes [4]. Other studies illustrated the biosynthetic potential of *Beauveria* spp. to produce diverse secondary metabolites, encompassing sesquiterpenes, steroids, tricyclic diterpenoids, cyclodepsipeptides, and various alkaloids [5,6]. Notably, compounds like beauverolides and beauvericins are known for their multifaceted roles as insecticidal, cytotoxic, and antimicrobial agents [4,7]. Similarly, the tenellins, polyketide synthase/nonribosomal-peptide synthetase (PKS-NRPS) products, originally isolated from *B. bassiana*, have been extensively studied regarding their biosynthesis [8]. However, only a limited number of studies have explored the biological functions of these secondary metabolites, and yet the full potential of these fungi remains largely untangled.

The knowledge gap extends to the complex interplay of fungal secondary metabolites and their usage in fighting the rapid increase of microbial resistance. For instance, biofilms formed by microbes often play a key role during chronic infections as they reduce the effectiveness of antibiotics, resulting in the overall increase of resistance. Nonetheless, no effective treatment for biofilm formation has been developed so far [9]. 

During our ongoing search for novel biofilm inhibitors, we investigated the production of secondary metabolites by the entomopathogenic fungus *B. neobassiana* from Thailand. The results are reported in the following narrative.

## 2. Materials and Methods

### 2.1. General Experimental Procedures

Optical rotations were measured at 20 °C using an MCP 150 circular polarimeter (Anton Paar, Seelze, Germany). UV/Vis spectra were collected with a Shimadzu UV2450 spectrophotometer (Shimadzu^®^, Kyoto, Japan). The optical rotation and UV/Vis spectra of all isolated secondary metabolites were measured in MeOH (Uvasol^®^, Merck, Darmstadt, Germany).

The 1D and 2D nuclear magnetic resonance (NMR) spectra of isolated compounds were obtained using an Avance III 700 spectrometer with a 5 mm TCI cryoprobe (Bruker Daltonics^®^, Bremen, Germany), ^1^H NMR: 700 MHz, ^13^C: 175 MHz, and an Avance III 500 spectrometer (Bruker, ^1^H NMR: 500 MHz, ^13^C: 125 MHz). Chemical shifts δ were referenced using the solvents DMSO-*d*_6_ or acetone-*d*_6_.

Analytical HPLC chromatograms and electrospray ionization mass spectra (ESI-MS) were acquired using an UltiMate 3000 Series UPLC (Thermo Fischer Scientific^®^, Waltham, MA, USA) equipped with a C18 column (Acquity UPLC BEH 2.1 × 50 mm, 1.7 µm; Waters, Milford, MA, USA) and an amaZon speed ESI-Iontrap-MS (Bruker) using a sample injection volume of 2 µL and a flow rate of 0.6 mL/min. A gradient elution was applied using a mobile phase consisting of solvent A (H_2_O + 0.1% formic acid (*v*/*v*)) and solvent B (MeCN + 0.1% formic acid (*v*/*v*)). The gradient started at 5% B, gradually increasing to 100% B over 20 min, followed by a 10 min hold at 100% B and UV-Vis detection in the range from 190 to 600 nm.

High-resolution electrospray ionization mass spectra (HR-ESI-MS) were obtained using an Agilent 1200 Infinity Series HPLC-UV system (Agilent Technologies^®^, Santa Clara, CA, USA) equipped with a C18 Acquity UPLC BEH analytical column (2.1 × 50 mm, 1.7 µm; Waters^®^, Milford, MA, USA) connected to a maXis^®^ Electrospray Time-of-flight mass spectrometer (ESI-TOF-MS; Bruker). The experimental conditions for acquiring the HR-ESI-MS data were identical to those used for ESI-MS.

### 2.2. Fungal Material

The strain *Beauveria neobassiana* Khons., Kobmoo & Luangsa-ard BCC 31604 was found growing on an adult unidentified beetle (Coleoptera) and collected and isolated on 22 July 2008, by a team including A. Khonsanit, J.J. Luangsa-ard, K. Tasanathai, P. Srikitikulchai, and S. Mongkolsamrit. The collection site was in Chiang Rai Province, Chiang Khong District, along the scenic Phlu Kaeng Waterfall Nature Trail, located at coordinates 20.26° N latitude and 100.39° E longitude. The herbarium material and the culture of the fungus are located in the collections of BIOTEC National Science and Technology Development Agency (NSTDA), Pathum Thani, Thailand, under the designation nos. BBH 23856 (specimen) and BCC 31604 (mycelial culture), respectively. The culture represents paratype material. The complete identification and characterization of the fungus was recently reported in an integrative phylogenomic study [4], in which mostly the molecular phylogenetic data served for segregation of the new species. 

### 2.3. Cultivation, Extraction and Isolation

*Beauveria neobassiana* BCC 31604 was cultured on potato dextrose agar (PDA; Fisher Scientific GmbH, Schwerte, Germany) plates for 7 days. A total of 5 mycelial plugs were carefully removed using a 7 mm diameter cork borer. The plugs were transferred into 500 mL Erlenmeyer flasks, each containing 200 mL of potato dextrose broth medium (PDB; 24 g/L; Fisher Scientific GmbH). For the main cultures, 50 flasks were used in total (10 L). The cultures were then incubated at 23 °C and 140 rpm. The glucose level was routinely monitored using urine glucose test strips (Dirui Industrial Co., Ltd., Changchun, China). The cultures were then harvested three days after the glucose was depleted. The mycelia were separated from the supernatant by vacuum filtration, and extracted separately.

A liquid–liquid extraction was conducted twice for the supernatant using ethyl acetate (1:1). The resulting organic phases were combined, filtered through anhydrous sodium sulfate, and evaporated under vacuum, while the aqueous phase was discarded. The mycelia were extracted with acetone (2 × 1 L) and subjected to two rounds of ultra-sonic bathing for 30 min at 40 °C. The resultant suspensions were filtered, and the acetone phase was collected and evaporated under vacuum. The resulting semi-solid residue from mycelial extract was then dispersed in 100 mL of water and subjected to liquid-liquid extraction against ethyl acetate as previously described. The obtained total extracts from the mycelia (91 mg) and the supernatant (269 mg) were analyzed by LC-MS and thereafter purified by preparative HPLC.

Both the mycelial and the supernatant extracts of *B. neobassiana* were subjected to purification using a PLC 2250 preparative HPLC system (Gilson, Middleton, WI, USA) and a Gemini C18 (250 × 50 mm, 10 μm; Phenomenex^®^, Torrance, CA, USA) as the stationary phase and the following conditions as the mobile phase: solvent A: deionized water (H_2_O) + 0.1% formic acid; solvent B: acetonitrile (MeCN) + 0.1% formic acid; flow: 45 mL/min for the mycelial extract and 30 mL/min for the supernatant extract; collected fraction volume: 15 mL. For the supernatant extract (269 mg), a gradient elution was applied as follows: from 35% B to 100% B in 50 min, then holding the gradient at 100% for 15 min, resulting in the isolation of five pure compounds: **6** (2.5 mg, *t*_R_ = 36 min), **5** (9.8 mg, *t*_R_ = 39 min), **2** (7.5 mg, *t*_R_ = 48 min), **3** (1.5 mg, *t*_R_ = 57 min), and **4** (4.0 mg, *t*_R_ = 60 min). For the mycelial extract (91 mg), gradient elution was applied as follows: from 25% B to 100% B in 65 min and ended with isocratic elution at 100% for 10 min, affording three pure substances: **1** (1.2 mg, *t*_R_ = 41 min), **6** (1.2 mg, *t*_R_ = 51 min), and **5** (1.8 mg, *t*_R_ = 54 min).

### 2.4. Spectral Data

#### 2.4.1. Pretenellin C (**1**)

Yellow amorphous solid; UV/Vis (MeOH) λ_max_ (log ε) 332 nm (3.86), 249 nm (4.40), 200 nm (4.58); NMR data (^1^H: 500 MHz, ^13^C: 125 MHz, acetone-*d*_6_) see Table 1; HR-ESI-MS: *m*/*z* 246.0393 [M–H_2_O+H]^+^ (calcd. 246.0397 for C_12_H_8_NO_5_^+^), 264.0499 [M+H]^+^ (calcd. 264.0503 for C_12_H_10_NO_6_^+^).

#### 2.4.2. Prototenellin D (**2**)

Pale yellow amorphous solid; [*α*${]}_{D}^{20}$ –264° (*c* 0.01, MeOH); UV/Vis (MeOH) λ_max_ (log ε) 368 nm, 224 nm, 200 nm; NMR data (^1^H: 500 MHz, ^13^C: 125 MHz, DMSO-*d*_6_) comparable to the previously described spectral data [8]; HR-ESI-MS: *m*/*z* 354.1707 [M–H_2_O+H]^+^ (calcd. 354.1700 for C_21_H_24_NO_4_^+^), 372.1812 [M+H]^+^ (calcd. 372.1805 for C_21_H_26_NO_5_^+^), 394.1633 [M+Na]^+^ (calcd. 394.1625 for C_21_H_25_NNaO_5_^+^).

#### 2.4.3. Pretenellin B (**3**)

Bright yellow powder; UV/Vis (MeOH) λ_max_ (log ε) 346 nm, 230 nm; NMR data (^1^H: 500 MHz, ^13^C: 125 MHz, DMSO-*d*_6_) comparable to the previously described spectral data [8]; HR-ESI-MS: *m*/*z* 354.1700 [M+H]^+^ (calcd. 354.1700 for C_21_H_24_NO_4_^+^), 376.1521 [M+Na]^+^ (calcd. 376.1519 for C_21_H_23_NNaO_4_^+^).

#### 2.4.4. Tenellin (**4**)

Yellow solid powder; UV/Vis (MeOH) λ_max_ (log ε) 344 nm, 221 nm; NMR data (^1^H: 500 MHz) comparable to the previously described spectral data [8]; HR-ESI-MS: *m*/*z* 352.1550 [M–H_2_O+H]^+^ (calcd. 352.1543 for C_21_H_22_NO_4_^+^), 370.1656 [M+H]^+^ (calcd. 370.1649 for C_21_H_24_NO_5_^+^), 392.1477 [M+Na]^+^ (calcd. 392.1468 for C_21_H_23_NNaO_5_^+^).

#### 2.4.5. 15-Hydroxytenellin (**5**)

Yellow amorphous solid; UV/Vis (MeOH) λ_max_ (log ε) 344 nm, 250 nm, 210 nm; NMR data (^1^H: 500 MHz, ^13^C: 125 MHz, DMSO-*d*_6_) comparable to the previously described spectral data [8]; HR-ESI-MS: *m*/*z* 368.1490 [M–H_2_O+H]^+^ (calcd. 368.1492 for C_21_H_22_NO_5_^+^), 386.1598 [M+H]^+^ (calcd. 386.1598 for C_21_H_24_NO_6_^+^), 408.1416 [M+Na]^+^ (calcd. 408.1418 for C_21_H_23_NNaO_6_^+^).

#### 2.4.6. Pyridovericin (**6**)

Yellow oil; UV/Vis (MeOH) λ_max_ (log ε) 342 nm, 248 nm, 210 nm; NMR data (^1^H: 500 MHz, ^13^C: 125 MHz, DMSO-*d*_6_) comparable to the previously described spectral data [10]; HR-ESI-MS: *m*/*z* 352.1541 [M–H_2_O+H]^+^ (calcd. 352.1543 for C_21_H_22_NO_4_^+^), 370.1649 [M+H]^+^ (calcd. 370.1649 for C_21_H_24_NO_5_^+^), 392.1467 [M+Na]^+^ (calcd. 392.1468 for C_21_H_23_NNaO_5_^+^).

### 2.5. Biological Assays

All isolated compounds were assessed for their antimicrobial activity using a serial dilution assay over a concentration range from 67 to 0.5 µg/mL following the previously described protocols [11,12]. In brief, the antimicrobial activity of all isolated metabolites was assessed by determining their minimum inhibitory concentration (MIC) against a range of different pathogens, including five fungal species: *Candida albicans* [DSM 1665], *Mucor hiemalis* [DSM 2656], *Schizosaccharomyces pombe* [DSM 70572], *Rhodotorula glutinis* [DSM 10134], and *Wickerhamomyces anomalus* [DSM 6766], as well as various Gram-positive: *Staphylococcus aureus* [DSM 346], *Bacillus subtilis* [DSM 10], *Mycolicibacterium smegmatis* [ATCC 700084], and Gram-negative bacteria: *Acinetobacter baumanii* [DSM 30008], *Escherichia coli* [DSM 1116], *Chromobacterium violaceum* [DSM 30191], and *Pseudomonas aeruginosa* [PA14]. In our experiments, gentamicin served as the positive control against most bacteria, while nystatin acted as the positive control against all fungi. For specific microorganisms, namely *A. baumannii*, *B. subtilis*, and *M. smegmatis*, ciprofloxacin, oxytetracycline, and kanamycin were used as positive controls, respectively.

The cytotoxicity of isolated compounds was determined as previously described [11,12] over a concentration range between 1 and 37 µg/mL against two mammalian cell lines: mouse fibroblasts (L-929) and human endocervical adenocarcinoma (KB-3.1). For compounds that exhibited cytotoxic properties against the first two cell lines, we also evaluated their cytotoxicity against breast cancer (MCF-7) and lung cancer (A-549) cell lines. Epothilone B served as the positive control. Following 5 days of incubation, we determined the minimum concentration of the tested compounds required to achieve 50% growth inhibition, expressed as IC_50_ values.

The compounds were evaluated for their ability to inhibit *S. aureus* (DSM 1104) biofilm formation, following the established procedure [13]. Briefly, serially diluted compounds (125–7.8 µg/mL) were co-incubated with *S. aureus* in 96-well plates in CASO medium supplemented with 4% of glucose (see Supplementary Materials). Biofilm inhibition was assessed by using Crystal Violet staining after 21 h, with microporenic acid A serving as a positive control and methanol as solvent control [14].

## 3. Results

### 3.1. Isolation and Structure Elucidation of Secondary Metabolites

**Figure 1:**
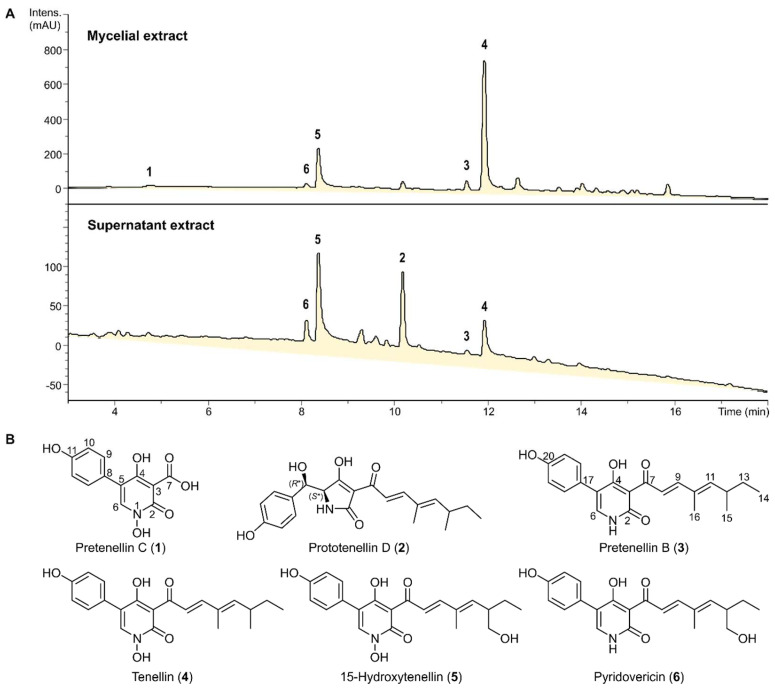
(**A**) HPLC−UV/Vis chromatograms (210 nm) of the crude extracts obtained from the mycelia and supernatant from the scaled-up fermentation of *B. neobassiana* in PDB medium. (**B**) Chemical structures of the isolated metabolites **1**–**6**.

Six secondary metabolites (**1**–**6**) were isolated after the scaled-up fermentation of *B. neobassiana* in PDB medium and subsequent purification of the obtained total extracts. As can be seen in Figure 1, the composition of the mycelial and culture filtrate extracts was similar, but the more lipophilic components **3** and **4** were preferentially located in the mycelial extracts. To establish their chemical structures, each of these compounds was analyzed using HR-ESI-MS, 1D- and 2D-NMR spectroscopy.

Compound **1** was obtained as a yellow amorphous solid, and its molecular formula was established as C_12_H_9_NO_6_ indicating nine degrees of unsaturation based on the HR-ESI-MS spectrum (Figure S2) revealing a protonated molecule at *m*/*z* 264.0499 [M+H]^+^ (calculated 264.0503). The ^1^H NMR spectrum of **1** (Table 1, Figure S3) revealed the presence of two proton resonances at δ_H_ 7.42 and δ_H_ 6.92, each with an integration index of 2, and both appeared as a doublet peak with an identical coupling constant (*J* value) of 8.3 Hz, indicating that compound **1** comprises a *para*-disubstituted aromatic ring. In addition, **1** also revealed two more deshielded singlet proton peaks at δ_H_ 8.18 directly correlated via the HSQC spectrum (Figure S6) to a deshielded olefinic carbon at δ_C_ 138.7, suggesting its presence in a heterocyclic aromatic ring and a second broad peak at δ_H_ 13.90 suggesting that **1** might comprise in its structure a carboxylic acid moiety. 

**Figure 2:**
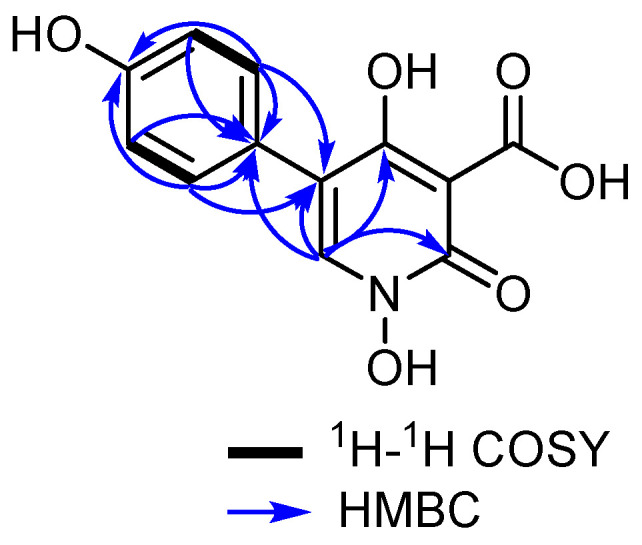
Key ^1^H-^1^H COSY and HMBC correlations of **1**.

By comparing the obtained results with the reported literature, compound **1** was tentatively identified as a 2-pyridone derivative [8]. To further confirm the depicted structure of **1**, its HMBC spectrum (Figure 2 and Figure S5) revealed key correlations from the deshielded aromatic proton at δ_H_ 8.18 (H-6) to four aromatic carbons distinguished into a carbonyl carbon (δ_C_ 161.2), an oxygenated aromatic carbon (δ_C_ 170.5), and two other unprotonated carbons (δ_C_ 123.6 and δ_C_ 115.3) that were assigned to C-2, C-4, C-5, and C-8, respectively. The HMBC spectrum of **1** (Figure 2 and Figure S5) revealed key correlations from two electromagnetically equivalent aromatic protons at δ_H_ 7.42 (d, *J* = 8.3 Hz, H-9/H-13) to a carbon resonance at δ_C_ 123.6 (C-4), confirming the presence of the phenyl moiety at C-4 of the 2-pyridone ring. By comparing the obtained ^1^H and ^13^C NMR data of **1** (Table 1) to those obtained and reported for pretenellin B (**3**) and 15-hydroxytenellin (**5**) [8], compound **1** was confirmed to feature N-OH and 3-carboxylic acid groups in its structure and hence to be a previously undescribed tenellin derivative that was trivially named as pretenellin C.

**Table 1:** ^1^H and ^13^C NMR data of **1**.

pos.	δ_H_^a^ (multi, *J* [Hz])	δ_C_, ^b^ Type
2		161.2, CO
3		98.2, C
4		170.5, CO
5		123.6, C
6	8.18 (s, 1H)	138.7, CH
7	13.94 (br)	n.d. ^c^
8		115.3, C
9,13	7.42 (d, 8.3, 2H)	131.2, CH
10,12	6.92 (d, 8.3, 2H)	115.9, CH
11		158.4, C

^a^ Measured in DMSO-*d*_6_ at 500 MHz. ^b^ Assigned based on HMBC and HSQC spectra. ^c^ n.d.: Not determined.

### 3.2. Biological Effects of Tenellin-Derived Metabolites

All isolated compounds were examined for their cytotoxic activity against two cell lines, L929 (fibroblast) and KB3.1 (ovary). The obtained results (Table 2) revealed that tenellin (**4**) was an equi- and the most potent against both cell lines with IC_50_ values of 0.79 µM. In addition, 15-hydroxytenellin (**5**) and pyridovericin (**6**) exhibitied less potent cytotoxic activities against the same cell lines with IC_50_ values ranging from 4.9 to 6.8 µM, while pretenellin C (**1**), prototenellin D (**2**) and pretenellin B (**3**) showed weak or no activity. Compounds **4**–**6** were further assessed against two additional cell lines namely, A-549 (lung) and MCF-7 (breast), where tenellin kept its leading potency with IC_50_ values of 0.24 and 2.0 µM, respectively, compared to epothilone B, a microtubule stabilizer first reported from the myxobacterium *Sorangium cellulosum* [15] and exhibited cytotoxic activity in paclitaxel-resistant tumor models [16]. Therefore, tenellin (**4**) with IC_50_ values down to low micromolar or nanomolar ranges could be noted as a promising scaffold to derivatize for developing a hit molecule for a cytotoxic lead compound.

All the isolated compounds were assessed for their antimicrobial activity against a panel of twelve microorganisms including Gram positive, Gram negative bacterial and fungal strains. The obtained results (Table 3) revealed that compounds **3**-**6** featured moderate to potent activities in particular against *S. aureus* and *B. subtilis* with tenellin (**4**) recognized as the most active at MIC values of 16.6 and 8.3 µg/mL, respectively.

**Figure 3:**
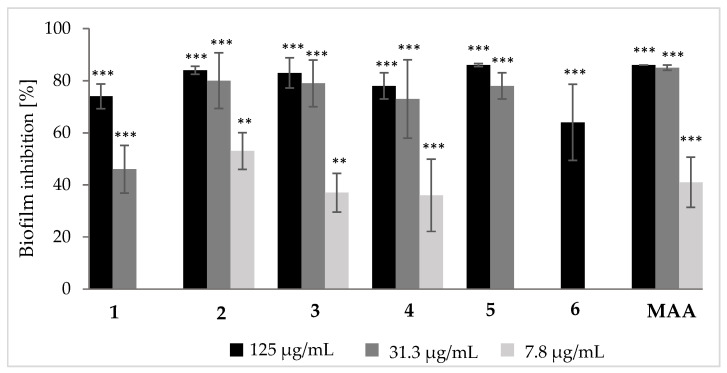
Inhibitory activity of compounds **1**–**6** and microporenic acid (MAA, positive control) on biofilm formation of *S. aureus* compared to the solvent control (MeOH, 0% biofilm inhibition). Error bars indicate SD of triplicates; *p* values: ** *p* < 0.01, *** *p* < 0.001. Differences between samples and the control group were determined by a two-tailed Student’s *t*-test. Statistical significance was defined as *p* < 0.01.

Furthermore, compounds **1**–**6** were evaluated for their antibiofilm properties against the pathogenic bacterium *S. aureus*. All tested compounds exhibited moderate inhibitory activities towards *S. aureus* (Figure 3, Table S1), with prototenellin D (**2**) being the most effective one. It inhibited the formation of *S. aureus* biofilms for more than 50% up to a concentration of 7.8 µg/mL. However, biofilm inhibition of pretenellin B (**3**) and tenellin (**4**) were also quite notable with activities of ca. 30% lasting till concentration of 7.8 µg/mL. Pretenellin C (**1**) and pyridovericin (**6**) were least active with activity loss below concentrations of 31.3 µg/mL and 62.5 µg/mL (Table S1), respectively.

## 4. Discussion

Secondary metabolites featuring the 2-pyridone moiety are previously reported from different sources with a wide range of biological activities as exemplified by furanpyridones [17], aspyridones [18] and ricinine [19]. Tenellins, 2-pyridone-containing compounds previously reported from *Beauveria bassiana* [8,10], have served as model compounds for investigating the biosynthesis of fungal metabolites. Exploring their biosynthetic pathway unveiled their respective biosynthetic gene clusters (BGCs) [8]. Remarkably, similar metabolites have been shown to exhibit a broad range of bioactivities including prominent neuritogenic and cytotoxic activities [20] in addition to their antimicrobial and antimalarial properties [21].

Herein, six tenellin derivatives were obtained from *B. neobassiana* distinguished into five known (**2**–**6**) alongside with one previously undescribed derivative, pretenellin C (**1**) whose closely related derivative, lacking the hydroxyl group at C-11, was patented in 2004 as an antiviral agent against hepatitis C virus [22].

In the cytotoxicity assay, tenellin (**4**) was the most potent suggesting a plausible role for both the 2-pyridone moiety and the aliphatic side chain in comparison to pretenellin C (**1**) and prototenellin D (**3**), respectively. The aliphatic side chain may impart higher lipophilicity, thus facilitating cell wall penetration. This notion was further supported by the observed 2- to 10-fold reduction in IC_50_ values for **5** and **6** compared to **4**, both featured an additional hydroxyl group at C-15 that might hinder cell penetration by raising the side chain polarity. On the contrary, pretenellin C (**1**) and prototenellin D (**2**), lacking the aliphatic side chain or 2-pyridone moiety, respectively, displayed no cytotoxicity that further supports the proposed structure-activity relationships. Intriguingly, the presence of N-hydroxylation, in **4** and **5** compared to **6**, did not reveal a notable influence on their cytotoxic effects.

In the antimicrobial activity assay, only compounds **3**–**6** revealed antimicrobial effects with tenellin (**4**) as the most active against *B. subtilis* and *S. aureus* at MICs of 8.3 and 16.6 µg/mL, respectively. Similarly, tenellin was reported in literature to possess antimicrobial activity against *S. aureus* (MIC = 6.25 µg/mL) and *Curvularia lunata* (MIC = 3.13 µg/mL) [21]. Compounds **3**, **5** and **6** revealed comparable activities against both tested microorganisms with MIC values of 66.6 µg/mL. By comparing their structures, it can be presumably deduced that N-hydroxylation of the 2-pyridone moiety in **4** resulted in a 4- to 8-fold increase in its antibacterial potency compared to **3** while the hydroxylation at C-15 as in **5** reduced antimicrobial activities against the same microorganisms and getting MIC values back to 66.6 µg/mL. 

In the biofilm inhibitory assay against *S. aureus*, prototenellin D (**2**), pretenellin B (**3**) and tenellin (**4**) revealed promising activities in a dose-dependent manner. However, the results obtained with 2-pyridone derivatives revealed that the aliphatic side chain at C-3 potentiates antibiofilm activity as demonstrated by tenellin (**4**) compared to pretenellin C (**1**). On the contrary, the hydroxylation at C-15 on of the side chain as in 15-hydroxytenellin (**5**), rather than tenellin (**4**), negatively affected the biofilm inhibitory activity over the tested concentration range. 

In conclusion, this study reports the results of chemical and biological prospection of tenellin derivatives from the entomopathogenic fungus *B. neobassiana*. The interpretation of these results suggested some preliminary structure-activity relationships (SARs) that might be useful for future development of antibiofilm agents, which in turn could be an alternative strategy to overcome multi-drug resistant human pathogens. Notably, for the biofilm inhibitors, the final goal could be to use them in combination with established antibiotics, in order to potentiate their activity because many antibiotics are not efficient enough when exposed to microbial biofilms. Future studies on this phenomenon may be rewarding. However, given the facts that (i) our present study is the first where a significant number of compounds have been tested simultaneously and (ii) it will most probably be necessary to employ semi-synthesis based on the availability of the tenellins in gram amounts, this would go far beyond the scope of the current work. 

## Figures and Tables

**Table 2:** Cytotoxicity (IC_50_) of compounds **1**–**6**.

	IC_50_ (µM)						Positive Control
Test Cell Line	1	2	3	4	5	6	Epothilone B (nM)
L929 (fibroblast)	n.a.	70.1	48.2	0.79	6.8	5.7	0.65
KB3.1 (ovary)	n.a.	64.7	20.2	0.79	6.0	4.9	0.17
A549 (lung)	n.d.	n.d.	n.d.	0.24	2.6	24.1	0.05
MCF-7 (breast)	n.d.	n.d.	n.d.	2.0	8.1	7.3	0.07

n.d.: not determined, n.a.: no effects up to 37 µg/mL.

**Table 3:** Minimum inhibitory concentration (MIC, µg/mL) of **1**–**6** against test organisms.

Test Microorganism	MIC (µg/mL)						Positive Control (µg/mL)
	1	2	3	4	5	6	
*Staphylococcus aureus*	n.i.	n.i.	66.6	16.6	66.6	66.6	0.21 ^G^
*Escherichia coli*	n.i.	n.i.	n.i.	n.i.	n.i.	n.i.	0.42 ^G^
*Bacillus subtilis*	n.i.	n.i.	66.6	8.3	66.6	66.6	16.6 ^O^
*Pseudomonas aeruginosa*	n.i.	n.i.	n.i.	n.i.	n.i.	n.i.	0.21 ^G^
*Wickerhamomyces anomalus*	n.d.	n.i.	n.d.	n.i.	n.i.	n.d.	16.6 ^N^
*Candida albicans*	n.i.	n.i.	n.i.	66.6	n.i.	n.i.	8.3 ^N^
*Acinetobacter baumannii*	n.d.	n.i.	n.d.	n.i.	n.i.	n.d.	0.52 ^C^
*Chromobacterium violaceum*	n.d.	n.i.	n.i.	n.i.	n.i.	n.d.	1.70 ^G^
*Schizosaccharomyces pombe*	n.d.	n.i.	n.d.	n.i.	n.i.	n.d.	4.20 ^N^
*Mucor hiemalis*	n.i.	n.i.	n.i.	66.6	n.i.	n.i.	8.30 ^N^
*Rhodotorula glutinis*	n.d.	n.i.	n.d.	n.i.	n.i.	n.d.	4.20 ^N^
*Mycobacterium smegmatis*	n.i.	n.i.	n.i.	n.i.	n.i.	n.i.	1.70 ^K^

n.i.: no inhibition up to 67 µg/mL. n.d.: not determined. G: Gentamycin; O: Oxytetracycline; N: Nystatin; C: Ciprofloxacin; K: Kanymycin.

## Data Availability

The DNA sequences are deposited in GenBank (https://www.ncbi.nlm.nih.gov/genbank/) and all other relevant data are included in the Supplementary Information.

## Supplementary Materials

The following supporting information can be downloaded at: https://www.mdpi.com/article/10.3390/jof10010069/s1, Figures S1–S35: HPLC chromatograms, LR-/HR-ESI-MS and 1D/2D NMR spectra of **1**–**6** in acetone-*d*_6_ or DMSO-*d*_6_ at 500 (for ^1^H) and 125 (for ^13^C) MHz. Table S1. Inhibition of biofilm formation of *S. aureus* by compounds **1**–**6** at different concentrations.
