# Supplementary material for: Bioprospection of Tenellins Produced by the Entomopathogenic Fungus Beauveria neobassiana
Source: J Fungi (Basel). 2024 Jan 15;10(1):69. doi: 10.3390/jof10010069 (PMC10821025; doi:10.3390/jof10010069)
Supplement: Supplementary file 1 [file jof-10-00069-s001.zip › jof-2746122-supplementary.pdf]

Supplementary Materials for

Bioprospection of Tenellins Produced by the Entomopathogenic Fungus *Beauveria neobassiana*

Rita Toshe [1,2], Esteban Charria-Girón [1,2], Artit Khonsanit [4], J. Jennifer Luangsa-ard [4], Syeda J. Khalid [1, 3], Hedda Schrey [1,2], Sherif S. Ebada [1,4,]*, and Marc Stadler [1,2,]*

[1] Department of Microbial Drugs, Helmholtz Centre for Infection Research GmbH (HZI), Inhoffenstraße 7, 38124 Braunschweig, Germany; rita.toshe@helmholtz-hzi.de; esteban.charriagiron@helmholtz-hzi.de

[2] Institut für Pharmazeutische Biologie, Universität des Saarlandes, Campus C2 3, 66123 Saarbrücken, Germany

[3] Institute of Microbiology, Technische Universität Braunschweig, Spielmannstraße 7, 38106 Braunschweig, Germany

[4] National Center for Genetic Engineering and Biotechnology (BIOTEC), National Science and Technology Development Agency (NSTDA), 111 Thailand Science Park, Phahonyothin Road, Khlong Nueng, Khlong Luang, Pathum Thani 12120, Thailand

[5] Department of Pharmacognosy, Faculty of Pharmacy, Ain Shams University, 11566 Cairo, Egypt

* Correspondence: sherif.elsayed@helmholtz-hzi.de; sherif_elsayed@pharma.asu.edu.eg (S.S.E.); Marc.Stadler@helmholtz-hzi.de (M.S.); Tel.: +49-531-6181-4240; Fax +49-531-6181-9499

Contents of Supporting Information

#	Contents	Page
**1**	Biofilm inhibition assay	**S3**
**2**	Table S1. Inhibition of biofilm formation of *S. aureus* by compounds **1-6** at different concentrations.	**S4**
**3**	Figure S1. LRESIMS spectra of **1**.	**S5**
**4**	Figure S2. HRESIMS spectra of **1**.	**S6**
**5**	Figure S3. ^{1}H NMR spectrum of **1** in acetone-d_6 at 500 MHz.	**S7**
**6**	Figure S4. ^{1}H-^{1}H COSY spectrum of **1** in acetone-d_6 at 500 MHz.	**S8**
**7**	Figure S5. HMBC spectrum of **1** in acetone-d_6 at 500 MHz.	**S9**
**8**	Figure S6. HSQC spectrum of **1** in acetone-d_6 at 500 MHz.	**S10**
**9**	Figure S7. ROESY spectrum of **1** in acetone-d_6 at 700 MHz.	**S11**
**10**	Figure S8. LRESIMS spectra of **2**.	**S12**
**11**	Figure S9. HRESIMS spectra of **2**.	**S13**
**12**	Figure S10. ^{1}H NMR spectrum of **2** in DMSO-d_6 at 500 MHz.	**S14**
**13**	Figure S11. ^{13}C NMR spectrum of **2** in DMSO-d_6 at 125 MHz.	**S15**
**14**	Figure S12. ^{1}H-^{1}H COSY spectrum of **2** in DMSO-d_6 at 500 MHz.	**S16**
**15**	Figure S13. HMBC spectrum of **2** in DMSO-d_6 at 500 MHz.	**S17**
**16**	Figure S14. HSQC spectrum of **2** in DMSO-d_6 at 500 MHz.	**S18**
**17**	Figure S15. ROESY spectrum of **2** in DMSO-d_6 at 700 MHz.	**S19**
**18**	Figure S16. LRESIMS spectra of **3**.	**S20**
**19**	Figure S17. HRESIMS spectra of **3**.	**S21**
**20**	Figure S18. ^{1}H NMR spectrum of **3** in DMSO-d_6 at 500 MHz.	**S22**
**21**	Figure S19. ^{1}H-^{1}H COSY spectrum of **3** in DMSO-d_6 at 500 MHz.	**S23**
**22**	Figure S20. HMBC spectrum of **3** in DMSO-d_6 at 500 MHz.	**S24**
**23**	Figure S21. HSQC spectrum of **3** in DMSO-d_6 at 500 MHz.	**S25**
**24**	Figure S22. LRESIMS spectra of **4**.	**S26**
**25**	Figure S23. HRESIMS spectra of **4**.	**S27**
**26**	Figure S24. ^{1}H NMR spectrum of **4** in DMSO-d_6 at 500 MHz.	**S28**
**27**	Figure S25. ^{1}H-^{1}H COSY spectrum of **4** in DMSO-d_6 at 500 MHz.	**S29**
**28**	Figure S26. LRESIMS of **5**.	**S30**
**29**	Figure S27. HRESIMS of **5**.	**S31**
**30**	Figure S28. ^{1}H NMR spectrum of **5** in DMSO-d_6 at 500 MHz.	**S32**
**31**	Figure S29. ^{1}H-^{1}H COSY spectrum of **5** in DMSO-d_6 at 500 MHz.	**S33**
**32**	Figure S30. HSQC spectrum of **5** in DMSO-d_6 at 500 MHz.	**S34**
**33**	Figure S31. HRESIMS of **6**.	**S35**
**34**	Figure S32. ^{1}H NMR spectrum of **6** in DMSO-d_6 at 500 MHz.	**S36**
**35**	Figure S33. ^{1}H-^{1}H COSY spectrum of **6** in DMSO-d_6 at 500 MHz.	**S37**
**36**	Figure S34. HMBC spectrum of **6** in DMSO-d_6 at 500 MHz.	**S38**
**37**	Figure S35. HSQC spectrum of **6** in DMSO-d_6 at 500 MHz.	**S39**

Biofilm inhibition assay

Biofilm inhibition assays were conducted according to the methodology previously described (Soliga *et al*., 2021), targeting the inhibition of biofilm formation in *Staphylococcus aureus* (DSM 1104).

S. aureus were cultured in LB and CASO medium at 37 °C for 18 h, respectively, in a shaker at 120 rpm after being thawed from a -20 °C stock solution. The culture's optical density at 600 nm (OD600) was measured and adjusted to a 0.1 McFarland standard in M63 minimal medium supplemented with necessary nutrients and CASO medium with 4% glucose for *S. aureus*, respectively. Microtiter plates were loaded with 150 µL of the bacterial suspension containing serially diluted test compounds (7.8 -125 µg/mL) and incubated for 24 h at 37 °C. The evaluation of biofilm inhibition was performed by staining the biofilm with 0.1% crystal violet (CV). After staining, the plates were washed, and the biofilm dissolved in 95% ethanol. The absorbance of the dissolved biofilm was measured at 530 nm using a plate reader. Negative control involved methanol (2.5%) while microporenic acid A at a concentration range of 7.8 to 125 µg/mL were used as the positive control for *S. aureus* (Chepkirui *et al*., 2018). All experiments were performed three times, and the standard deviation (SD) was maintained at or below 15% for *S. aureus*.

Differences between samples and the control group were determined by a two-tailed Student's *t*-test. Statistical significance was defined as $p < 0.01$. Analysis was carried out using GraphPad Prism 9® (GraphPad Software, San Diego, CA, USA).

References

Chepkirui, C.; Yuyama, KT.; Wanga, LA.; Decock, C.; Matasyoh, JC.; Abraham, WR.; Stadler, M. Microporenic acids A-G, biofilm inhibitors, and antimicrobial agents from the Basidiomycete *Microporus* Species. *J. Nat. Prod*. **2018**, 81, 778-784.

Soliga, KJ.; Bär, SI.; Oberhuber, N.; Zeng, H.; Schrey, H.; Schobert, R. Synthesis and bioactivity of ancorinoside B, A marine diglycosyl tetramic acid. *Mar. Drugs.* **2021**, 19, 583.

Table S1. Inhibition of biofilm formation of *S. aureus* by compounds **1-6** at different concentrations.

		Biofilm inhibition (% ±SD)						
**Tested organism**	**Conc.** (µg/ml)	**1**	**2**	**3**	**4**	**5**	**6**	**Microporenic acid A (MAA)**
S. aureus (DSM 1104)	125	74 ± 5	84 ± 17	83 ± 6	78 ± 5	86 ± 1	64 ± 14	86 ± 0
	62.5	63 ± 16	83 ± 9	83 ± 6	80 ± 4	85 ± 2	48 ± 5	84 ± 1
	31.25	46 ± 9	80 ± 10	79 ± 8	73 ± 15	78 ± 5	-	85 ± 1
	15.62	-	56 ± 8	52 ± 6	51 ± 19	47 ± 12	-	70 ± 13
	7.8	-	53 ± 7	37 ± 7	36 ± 13	-	-	41 ± 9

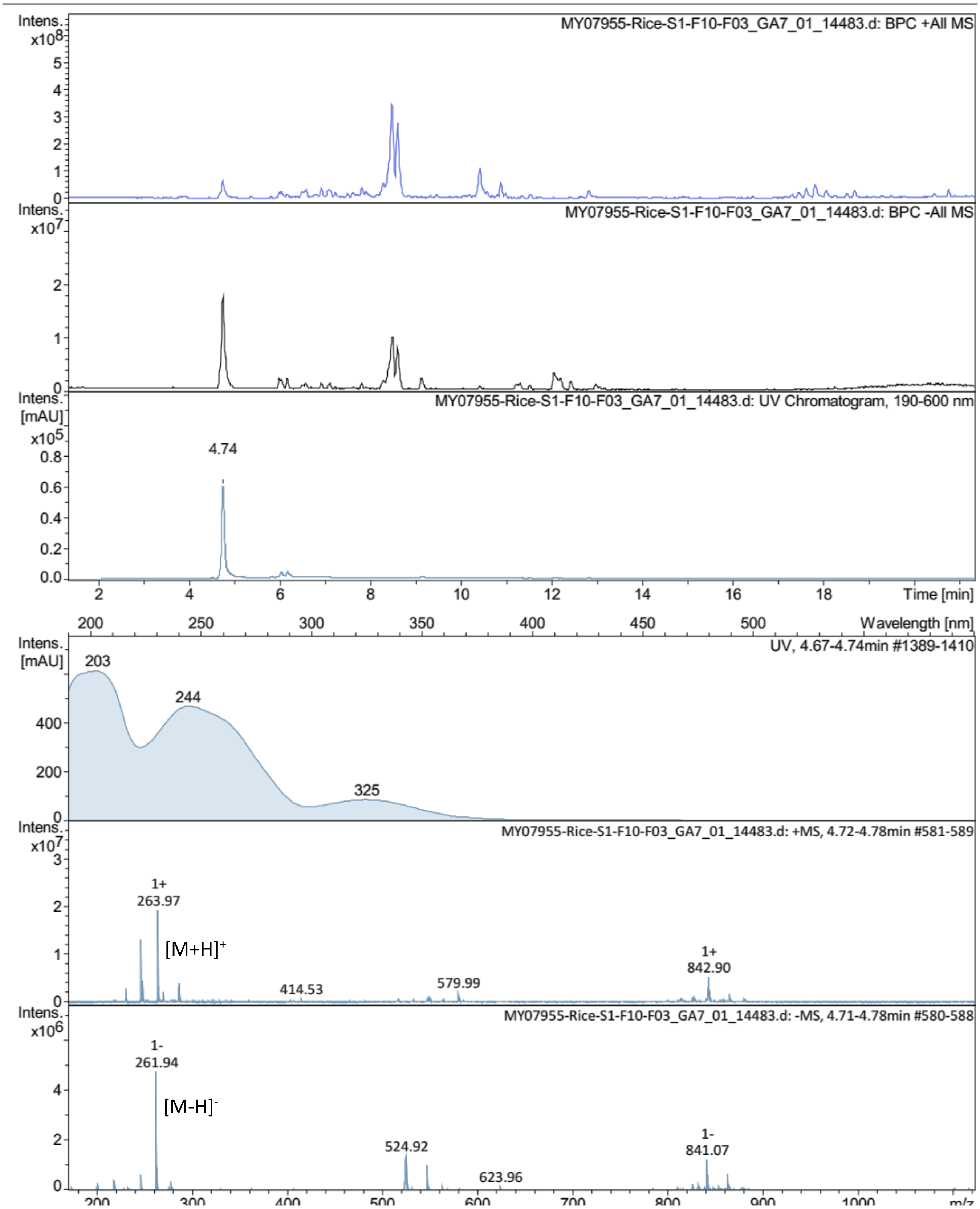

Bruker Compass DataAnalysis 4.4 printed: 21.06.2023 16:10:01 by: sel22 Page 1 of 1

Figure S1. LRESIMS of **1**.

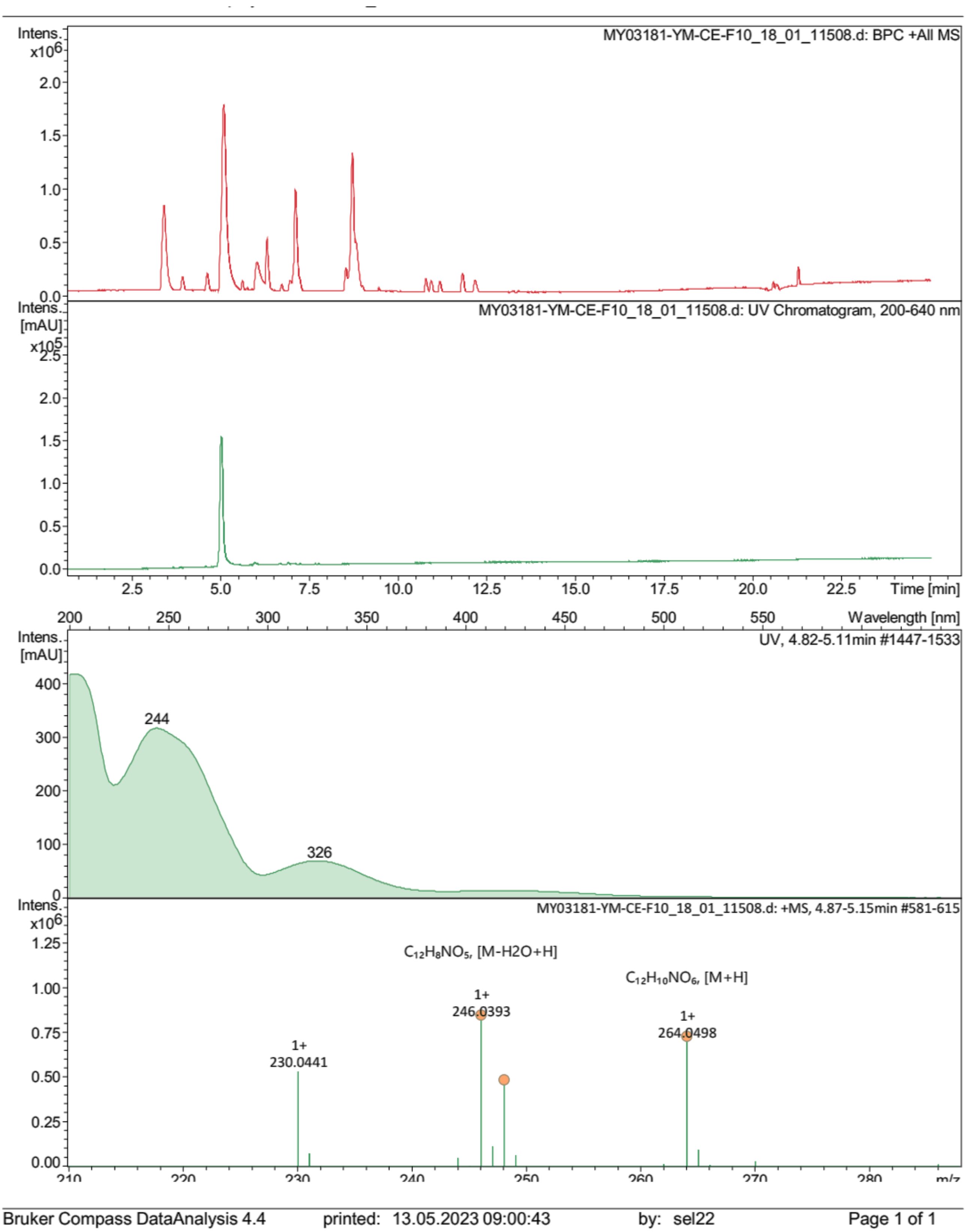

Figure S2. HRESIMS of **1**.

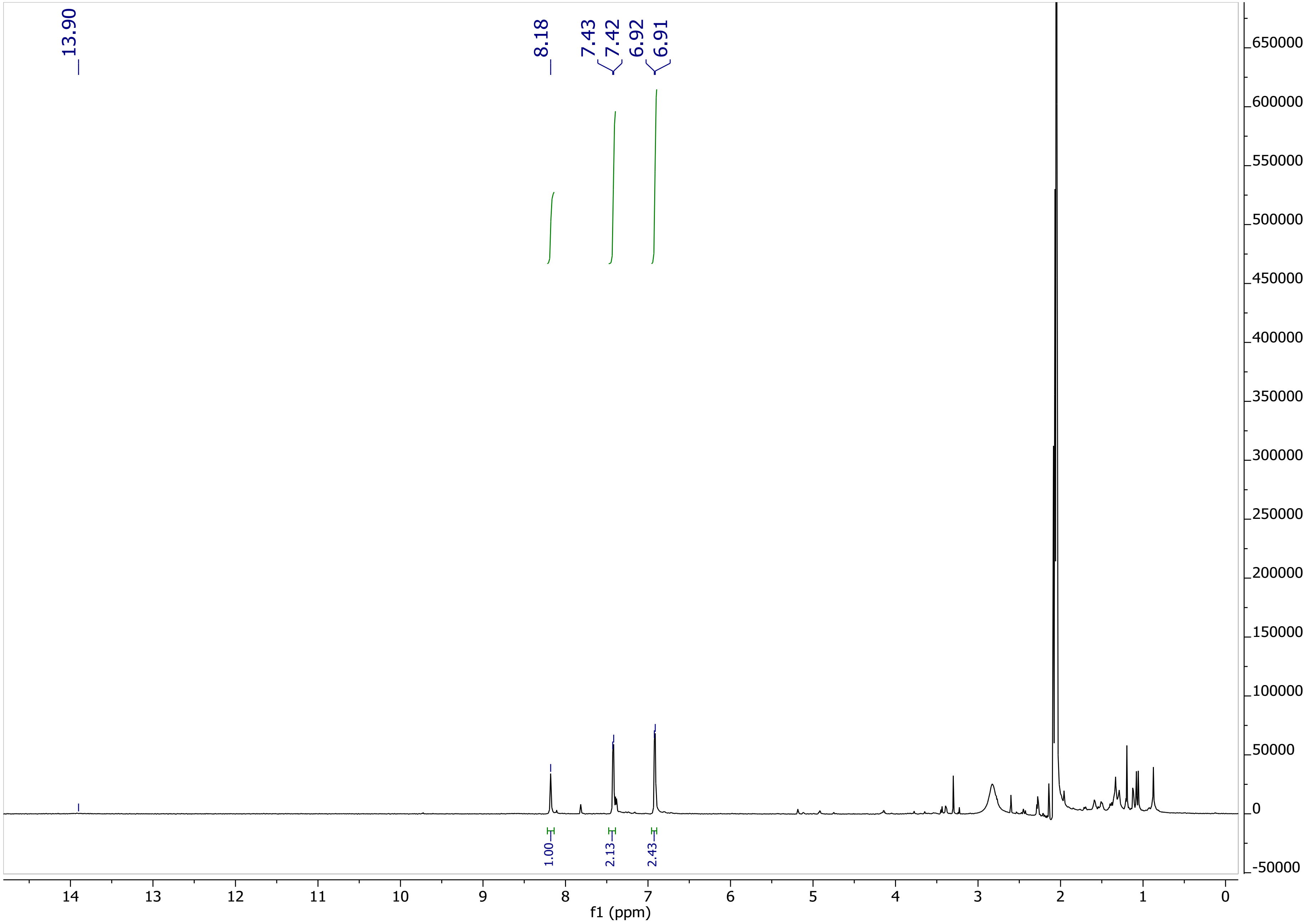

Figure S3. ^{1}H NMR spectrum of **1** in acetone-d_6 at 500 MHz.

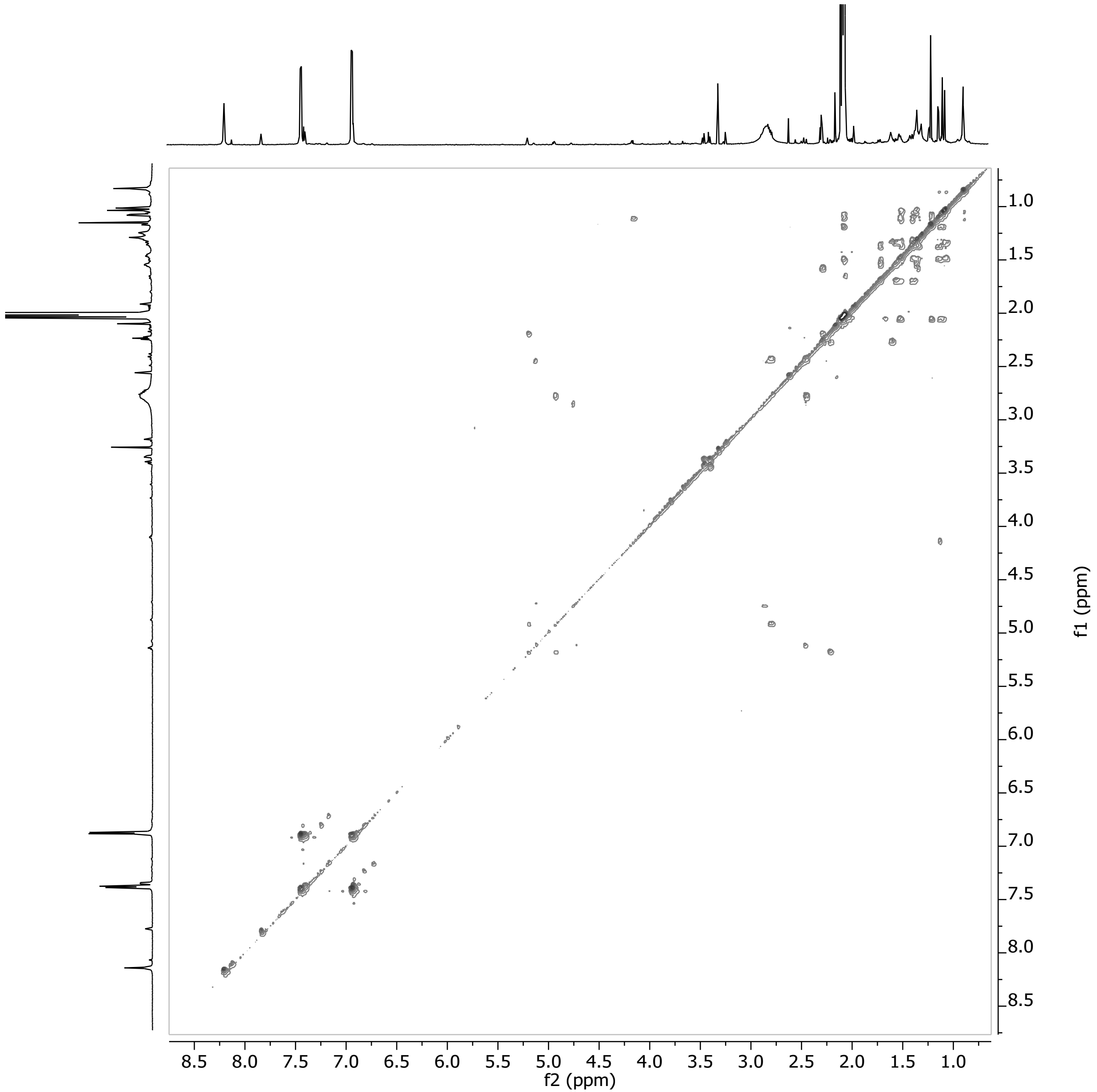

Figure S4. ^{1}H-^{1}H COSY spectrum of **1** in acetone-d_6 at 500 MHz.

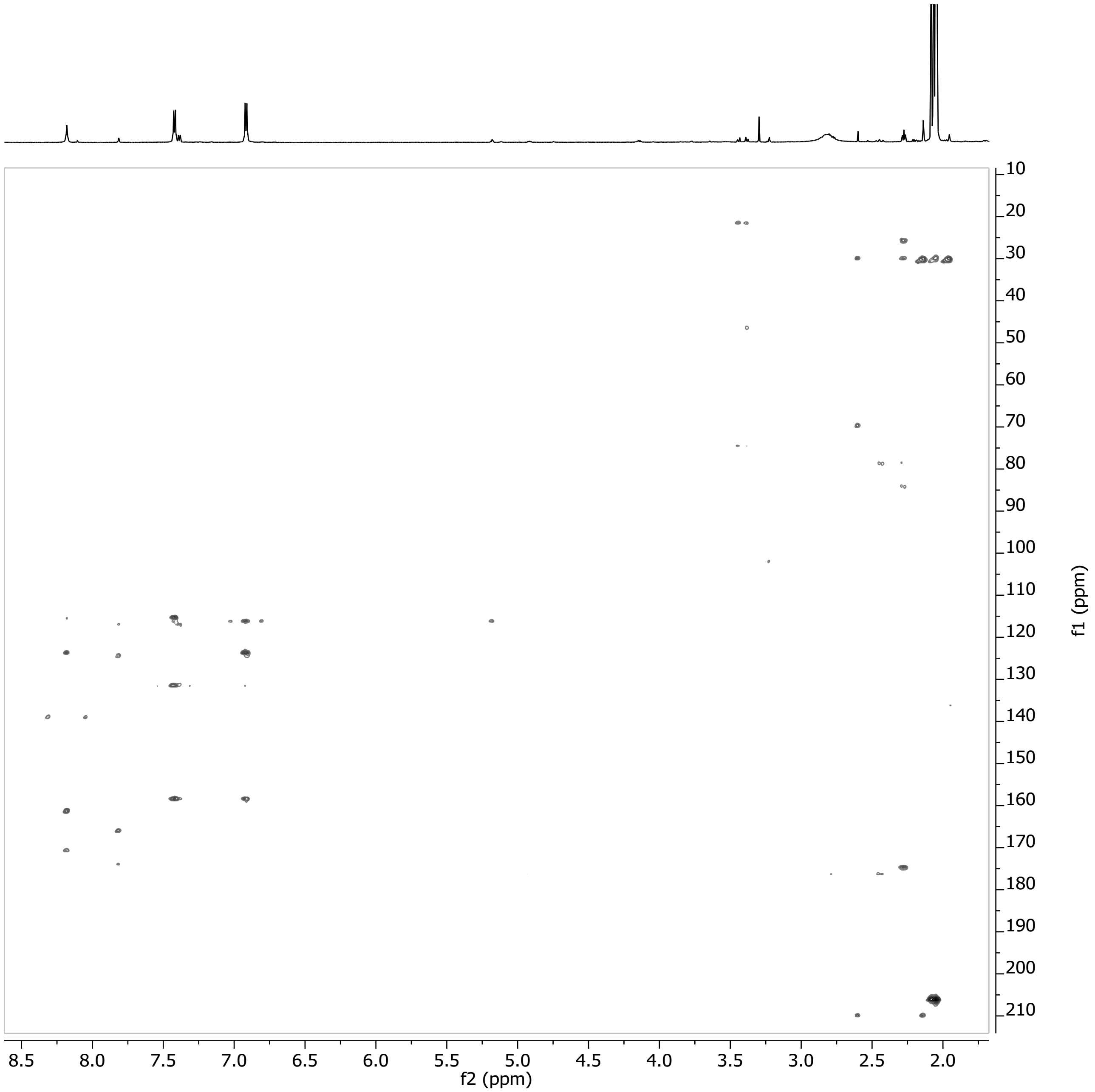

Figure S5. HMBC spectrum of **1** in acetone-d_6 at 500 MHz.

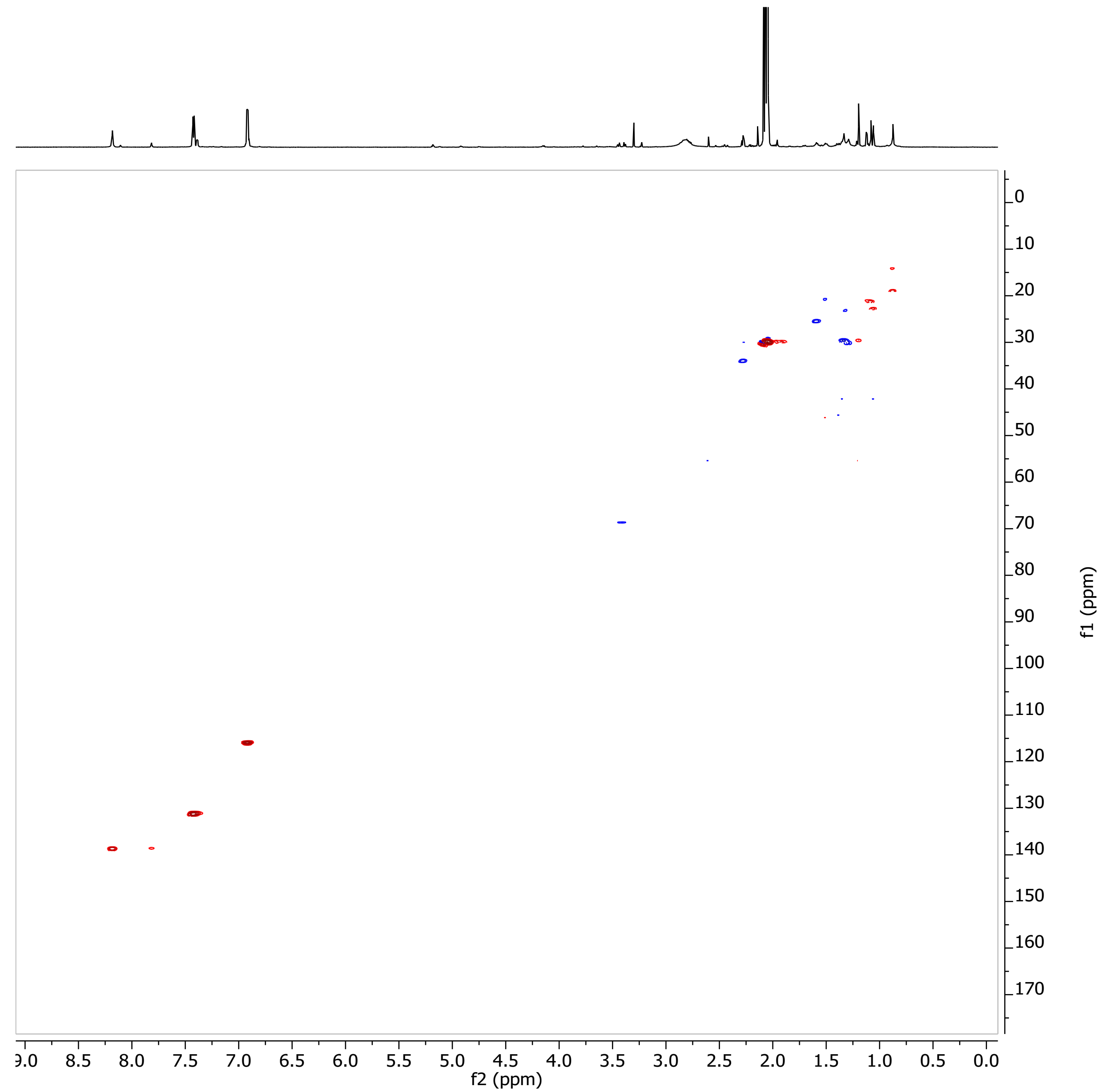

Figure S6. HSQC spectrum of **1** in acetone-d_6 at 500 MHz.

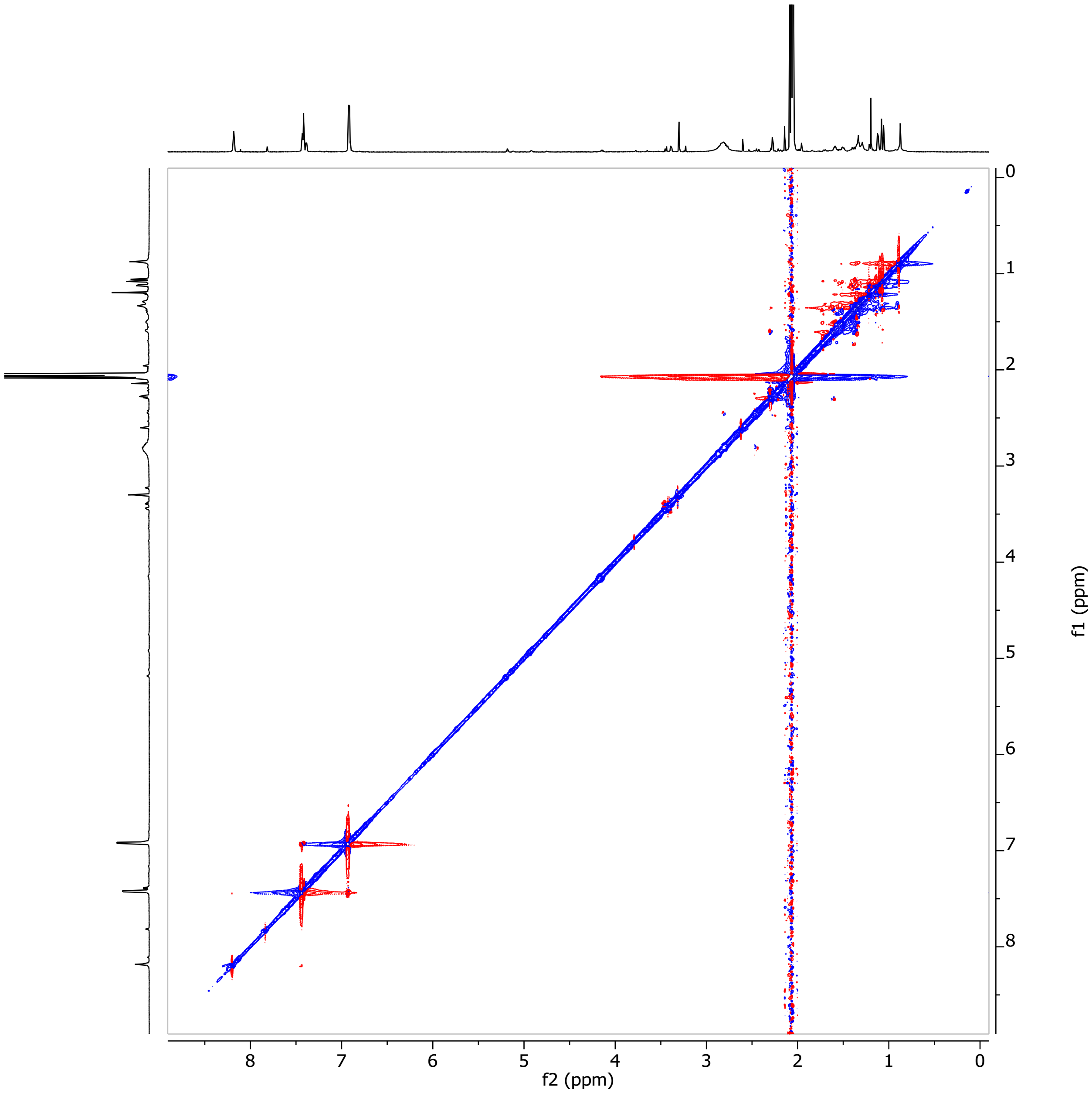

Figure S7. ROESY spectrum of **1** in acetone-d_6 at 700 MHz.

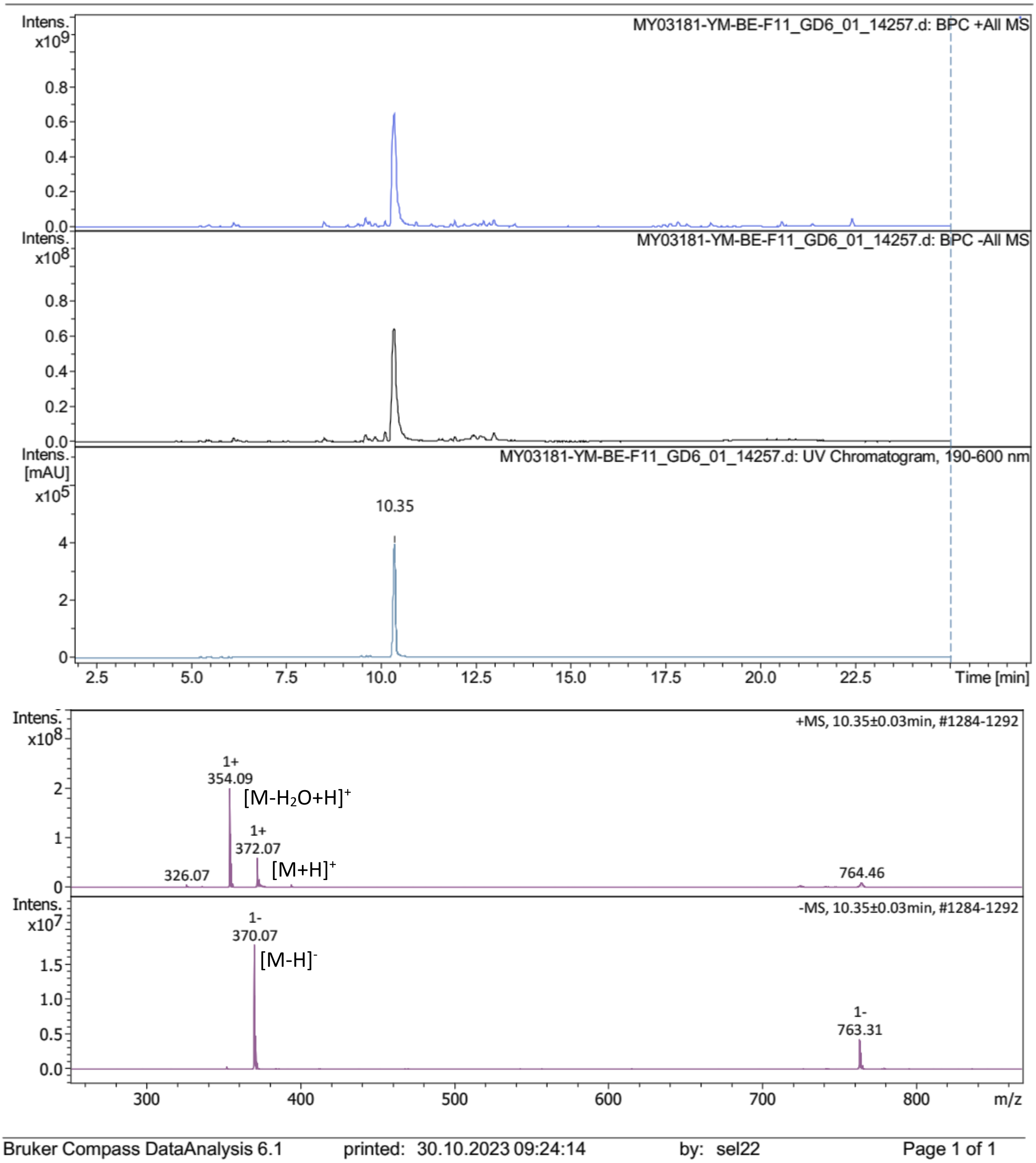

Figure S8. LRESIMS of **2**.

Generic Display Report

Analysis Info

Analysis Name	S:\DATA\MaXis\rto20_Rita Toshe\23_05\23_05_27\MY03181-YM-BE-F11_90_01_11713.d	Acquisition Date	28.05.2023 07:28:36
Method	pos_säure_10000_screening_ms_100_2500_line.m	Operator	ate06
Sample Name	MY03181-YM-BE-F11	Instrument	maXis
Comment	Screening01<br>Waters Acquity UPLC BEH C_18 1,7um 2.1x50mm		

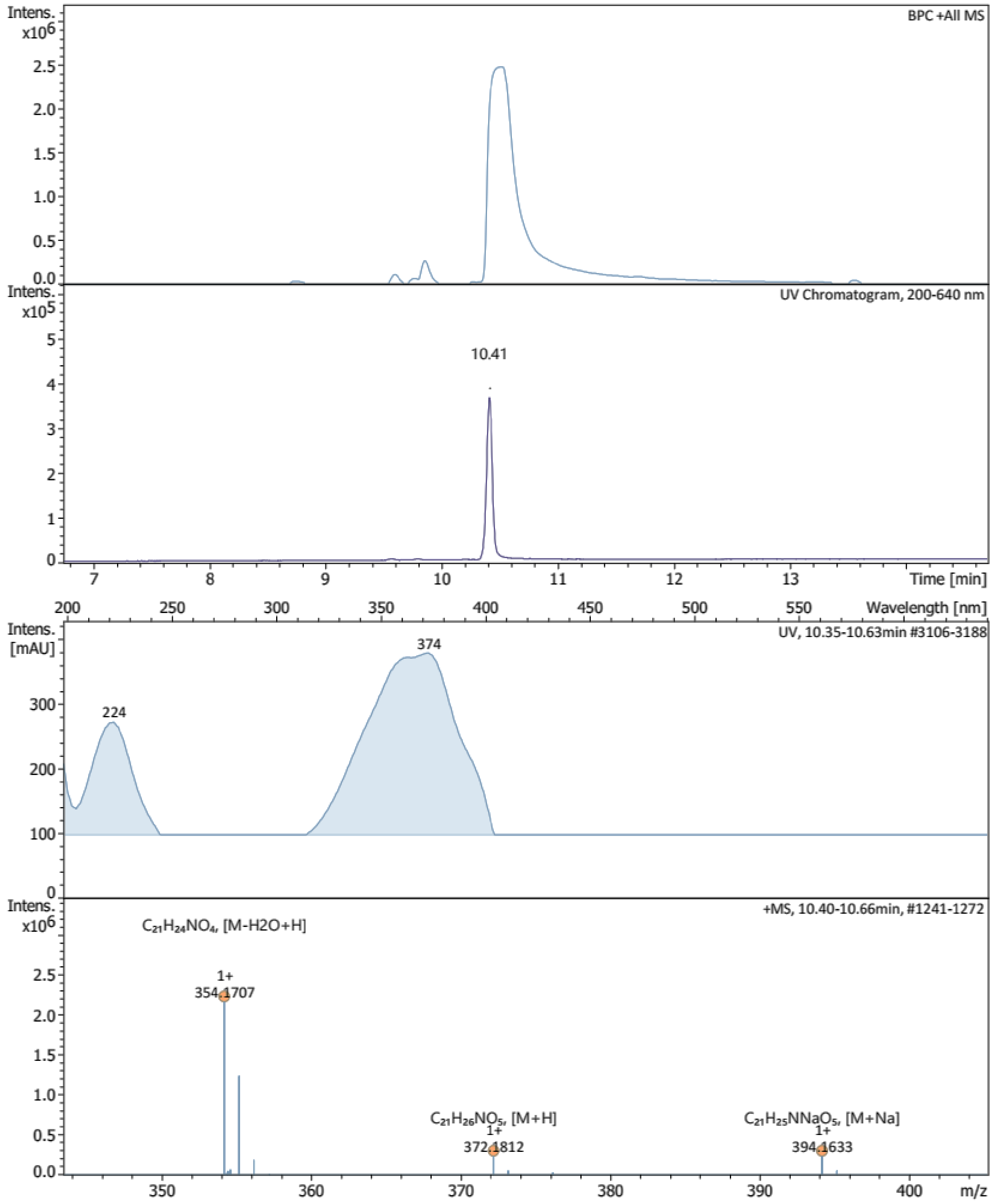

Bruker Compass DataAnalysis 6.1 printed: 30.10.2023 09:27:29 by: sel22 Page 1 of 1

Figure S9. HRESIMS of **2**.

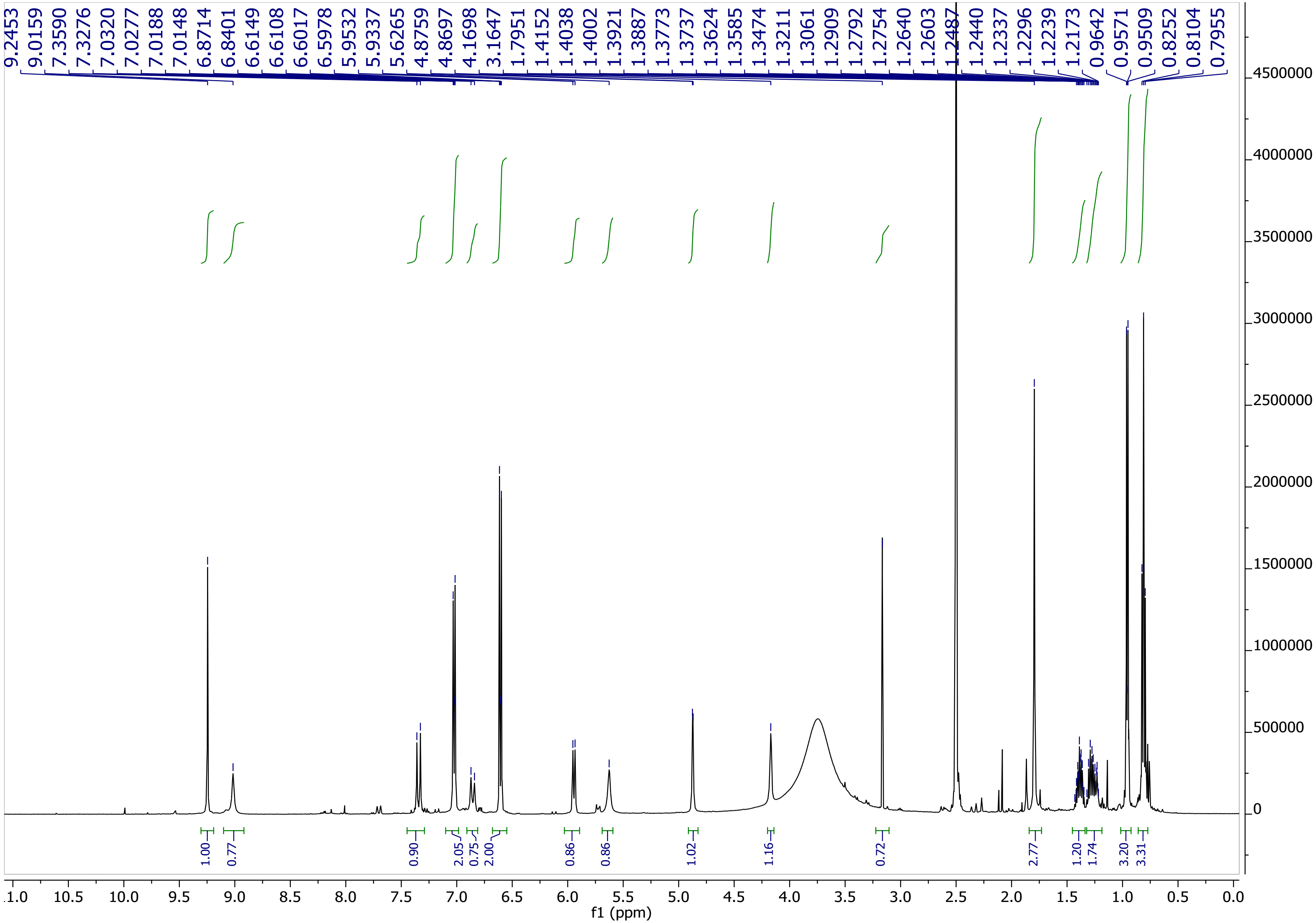

Figure S10. ^{1}H NMR spectrum of **2** in DMSO-d_6 at 500 MHz.

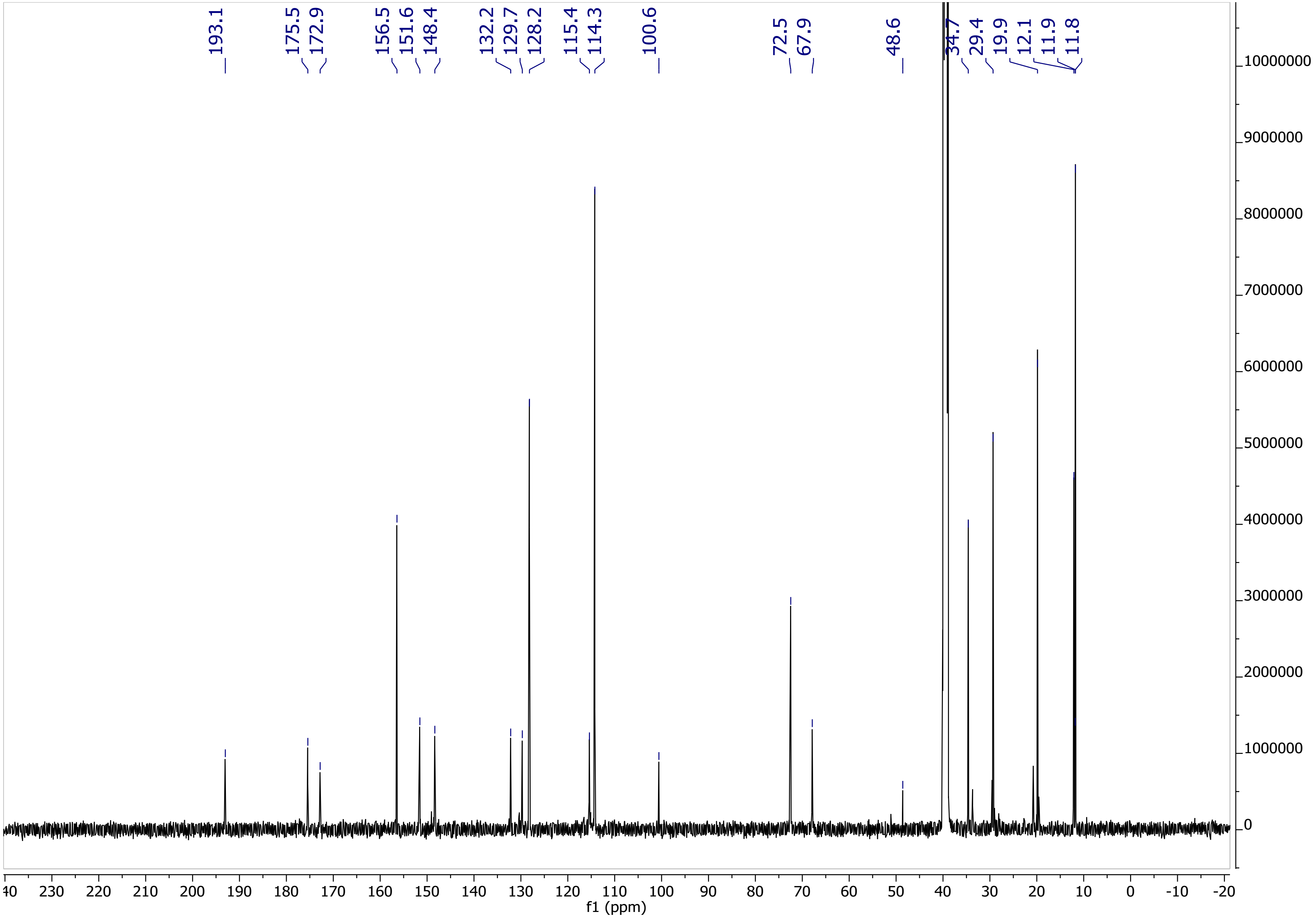

Figure S11. ^{1}H NMR spectrum of **2** in DMSO-d_6 at 125 MHz.

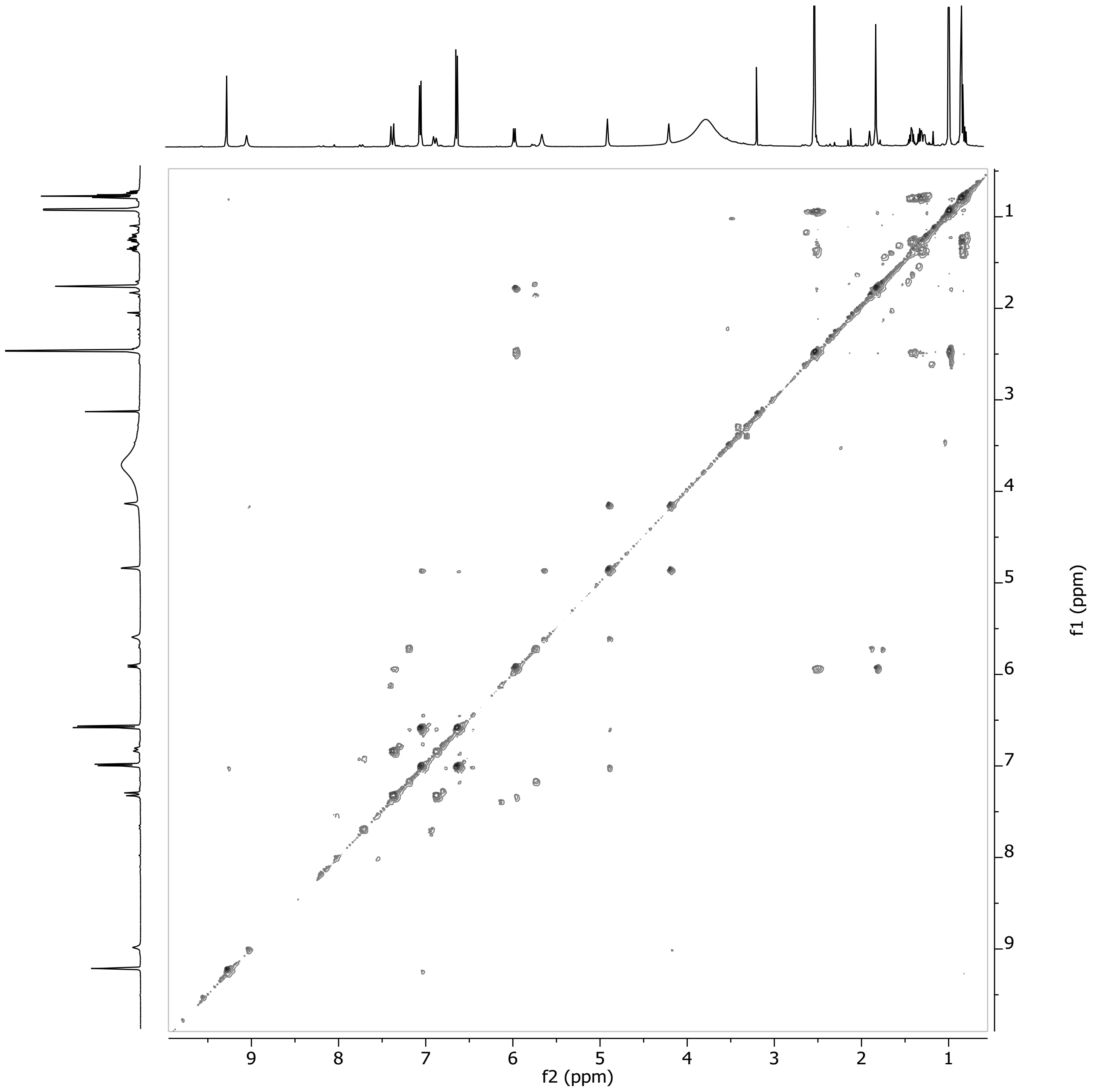

Figure S12. ^{1}H-^{1}H COSY spectrum of **2** in DMSO-d_6 at 500 MHz.

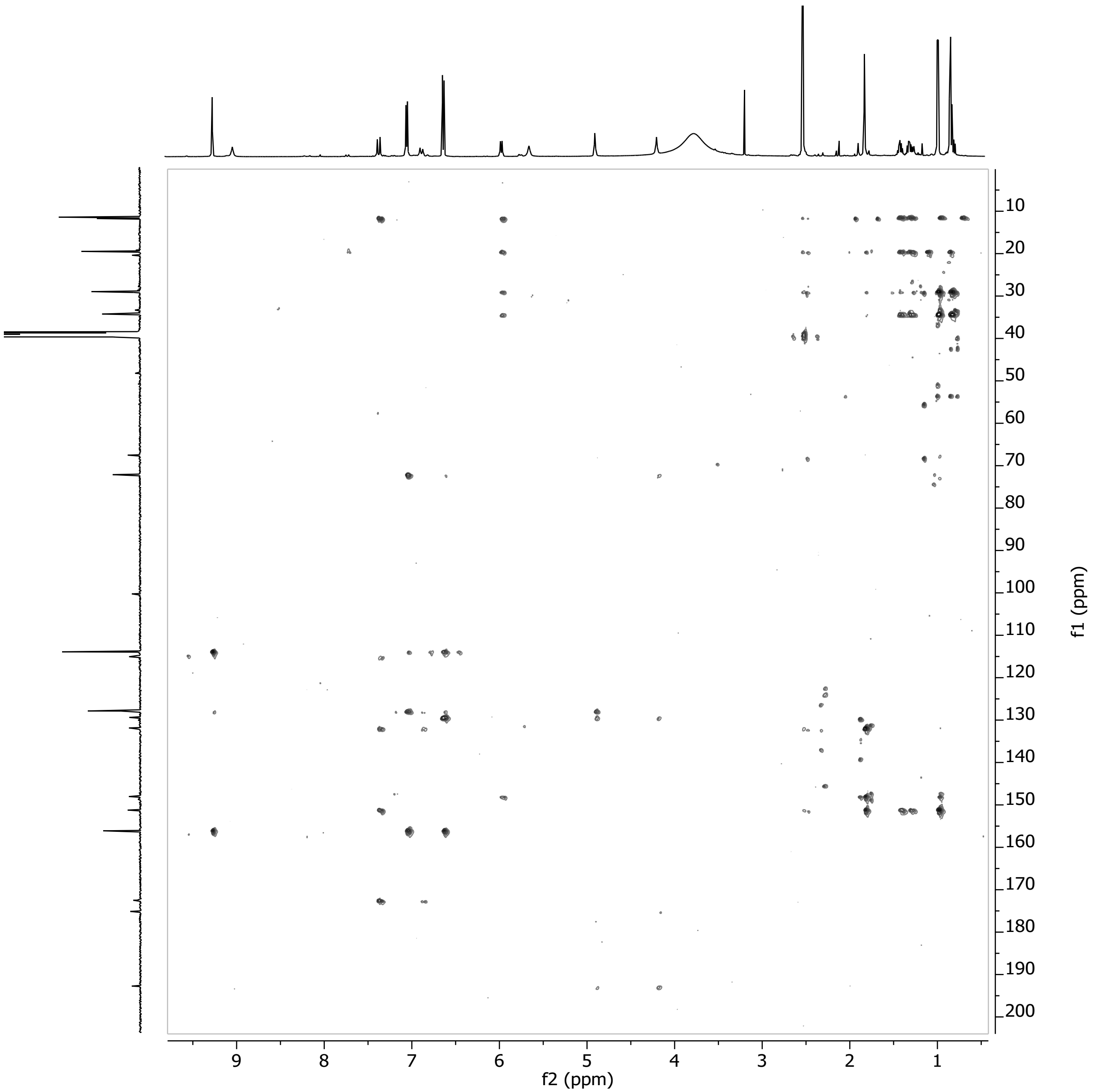

Figure S13. HMBC spectrum of **2** in DMSO-d_6 at 500 MHz.

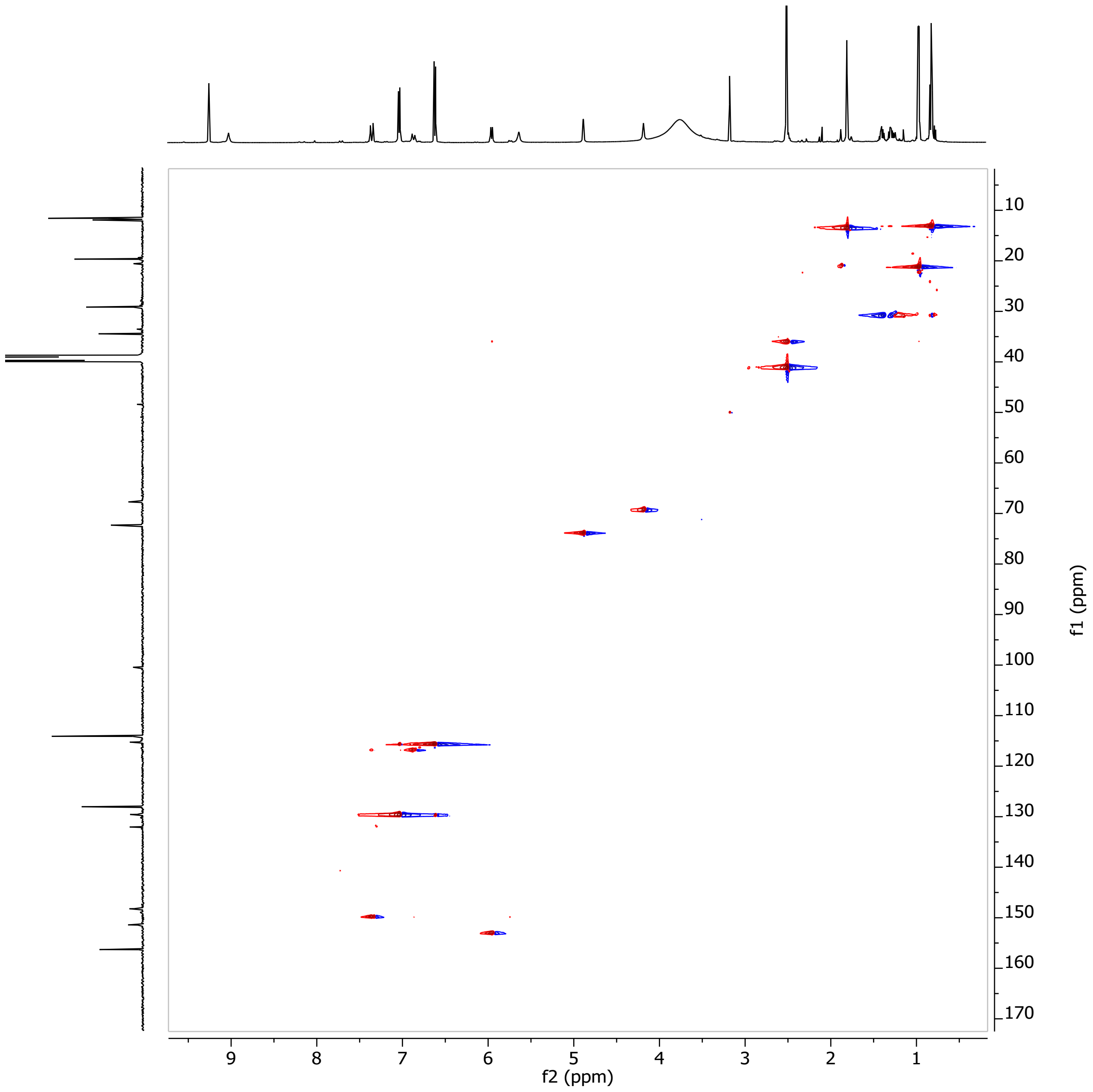

Figure S14. HSQC spectrum of **2** in DMSO-d_6 at 500 MHz.

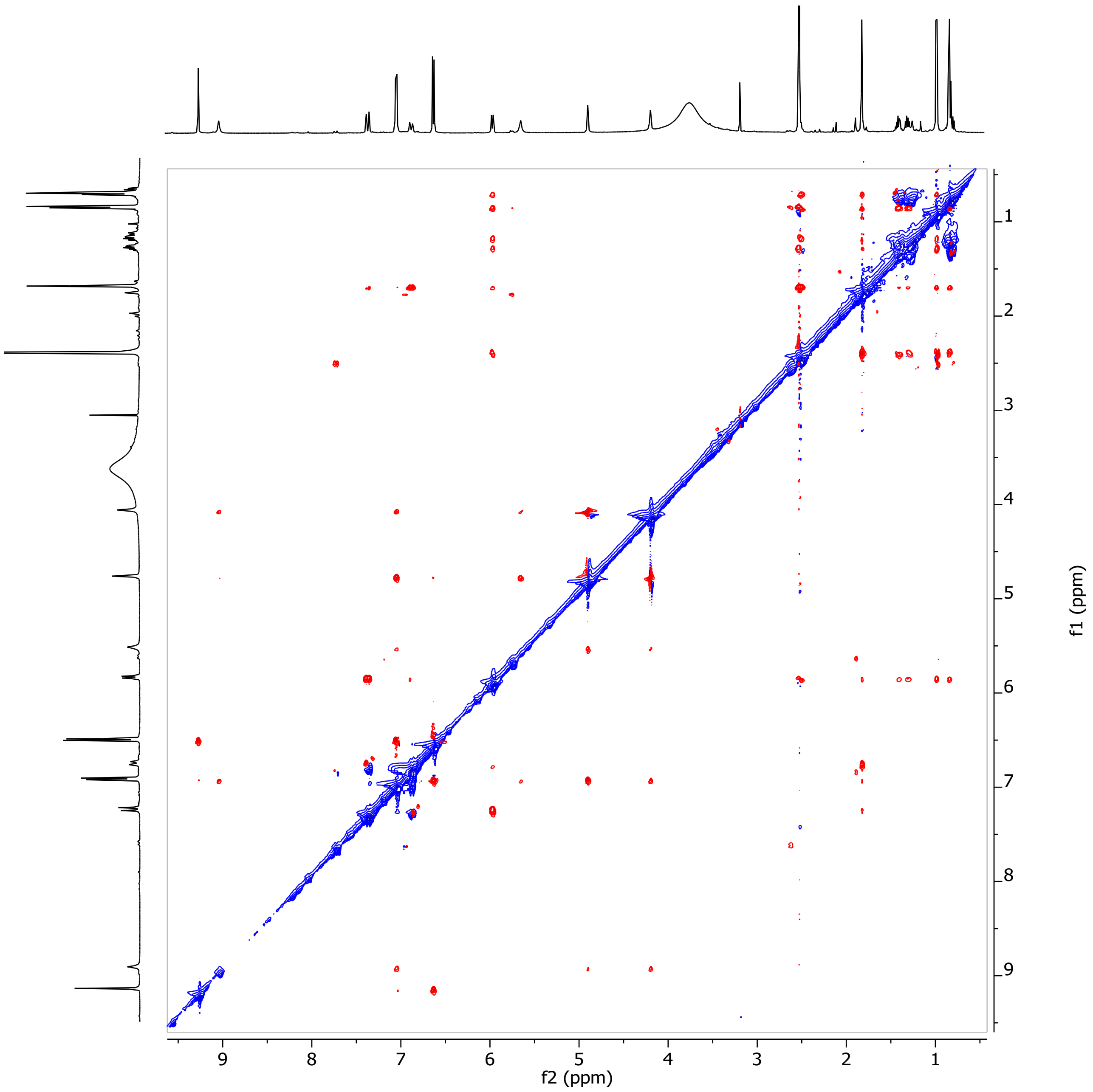

Figure S15. ROESY spectrum of **2** in DMSO-d_6 at 700 MHz.

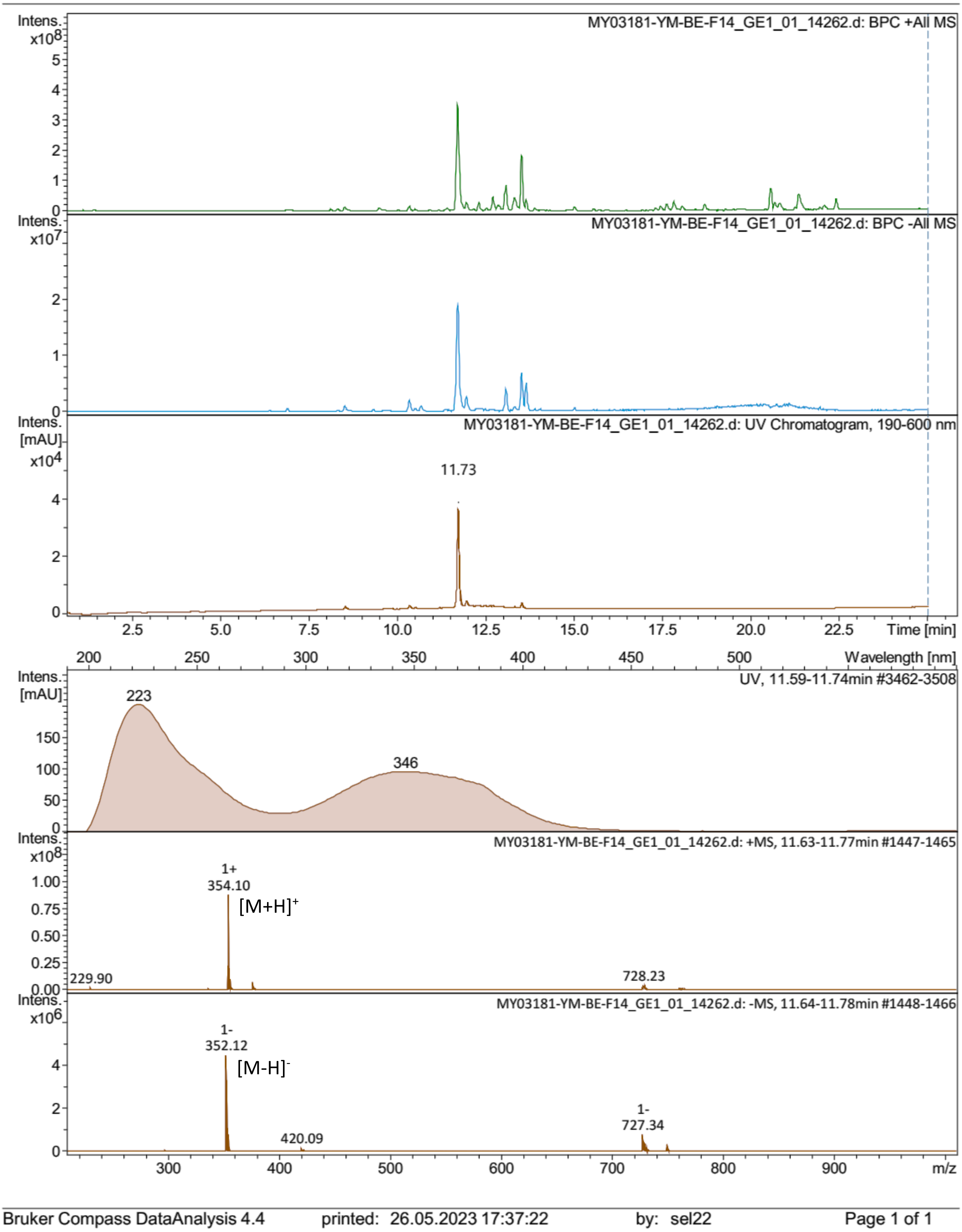

Figure S16. LRESIMS of **3**.

Generic Display Report

Analysis Info

		Acquisition Date	28.05.2023 09:01:25
Analysis Name	S:\DATA\MaXis\rto20_Rita Toshe\23_05\23_05_27\MY03181-YM-BE-F14_93_01_11716.d		
Method	pos_säure_10000_screening_ms_100_2500_line.m	Operator	ate06
Sample Name	MY03181-YM-BE-F14	Instrument	maXis
Comment	Screening01<br>Waters Acquity UPLC BEH C_18 1,7um 2.1x50mm		

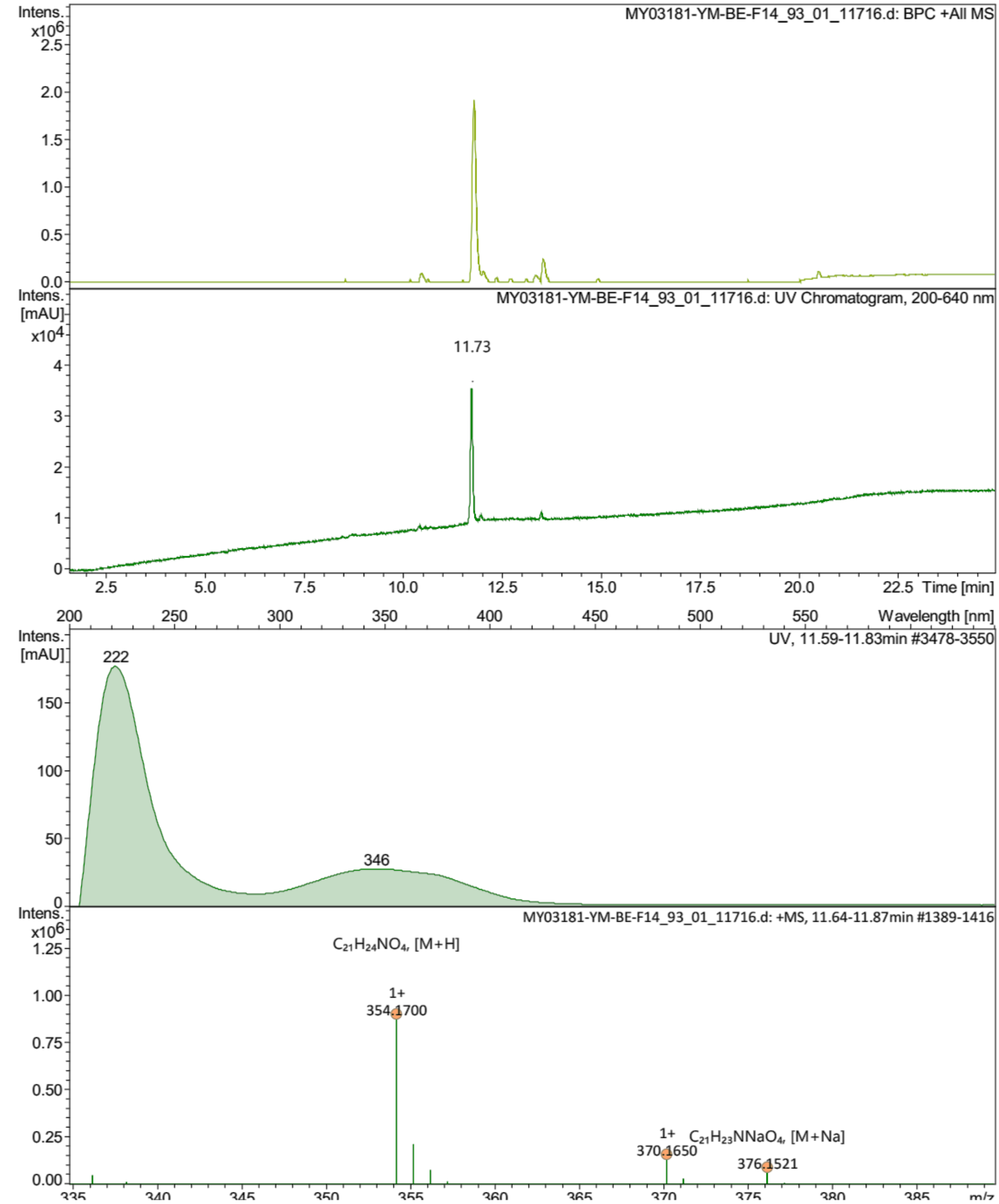

Bruker Compass DataAnalysis 4.4 printed: 30.05.2023 15:05:11 by: sel22 Page 1 of 1

Figure S17. HRESIMS of **3**.

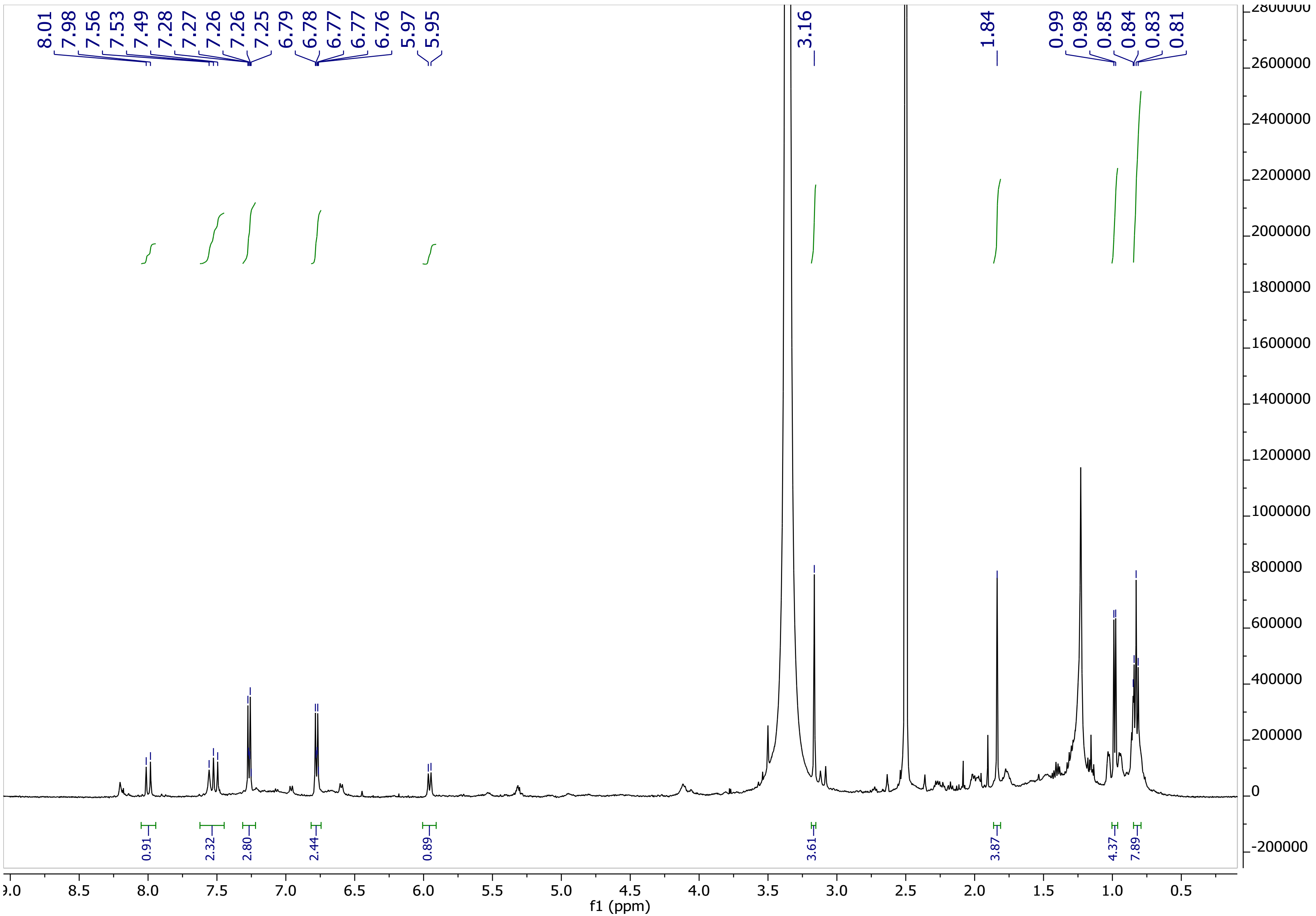

Figure S18. ^{1}H NMR spectrum of **3** in DMSO-d_6 at 500 MHz.

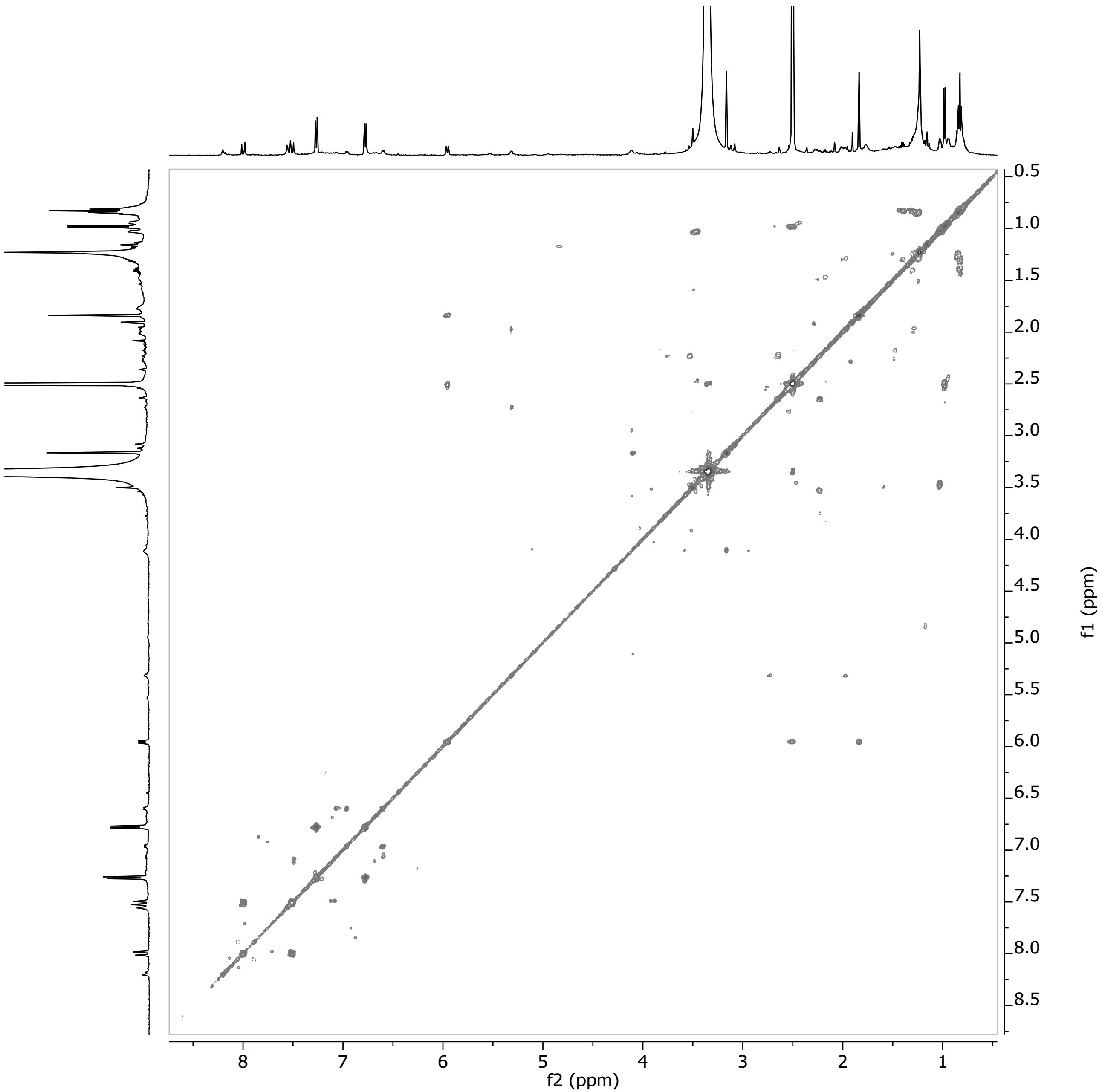

Figure S19. ^{1}H-^{1}H COSY spectrum of **3** in DMSO-d_6 at 500 MHz.

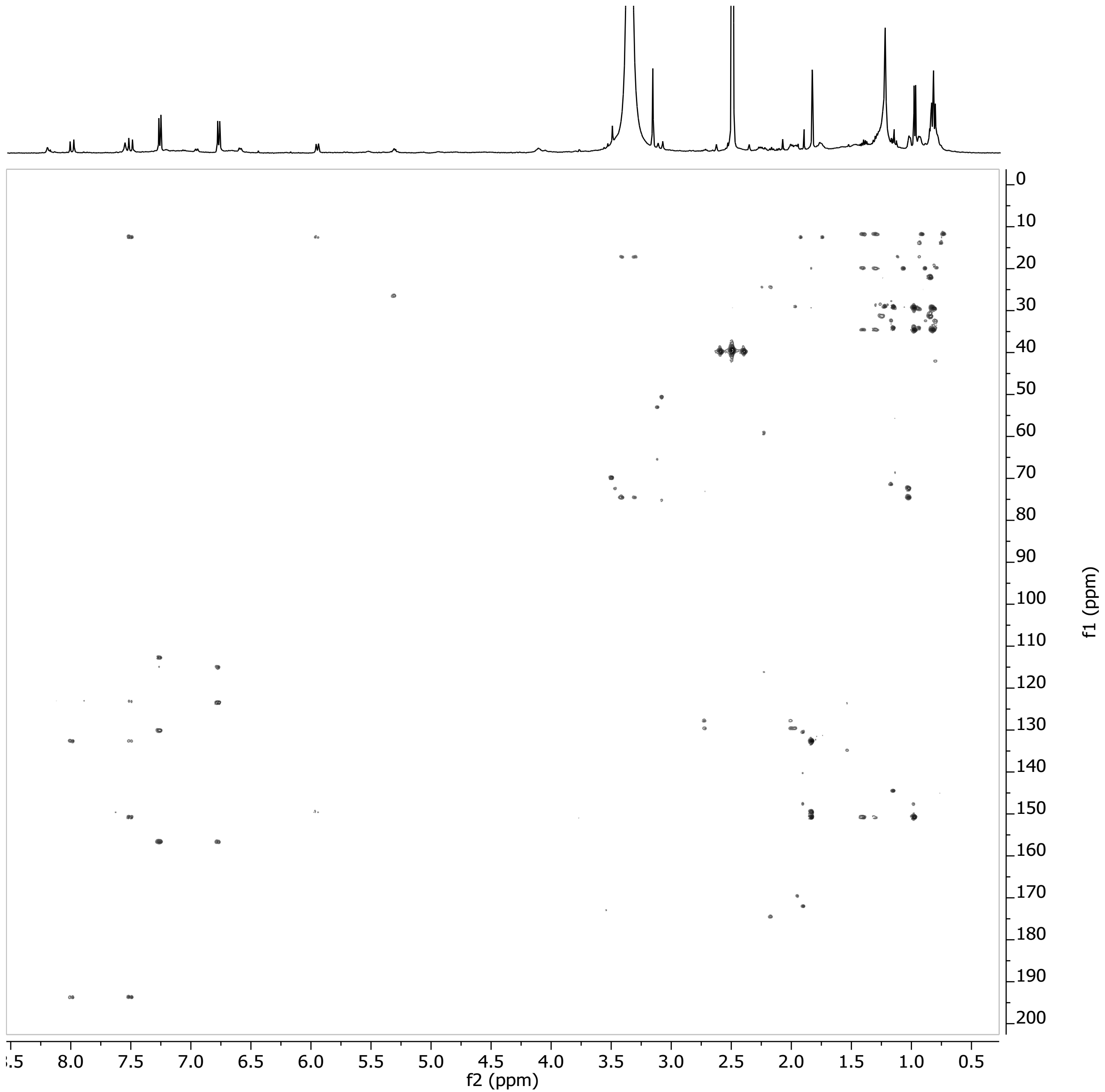

Figure S20. HMBC spectrum of **3** in DMSO-d_6 at 500 MHz.

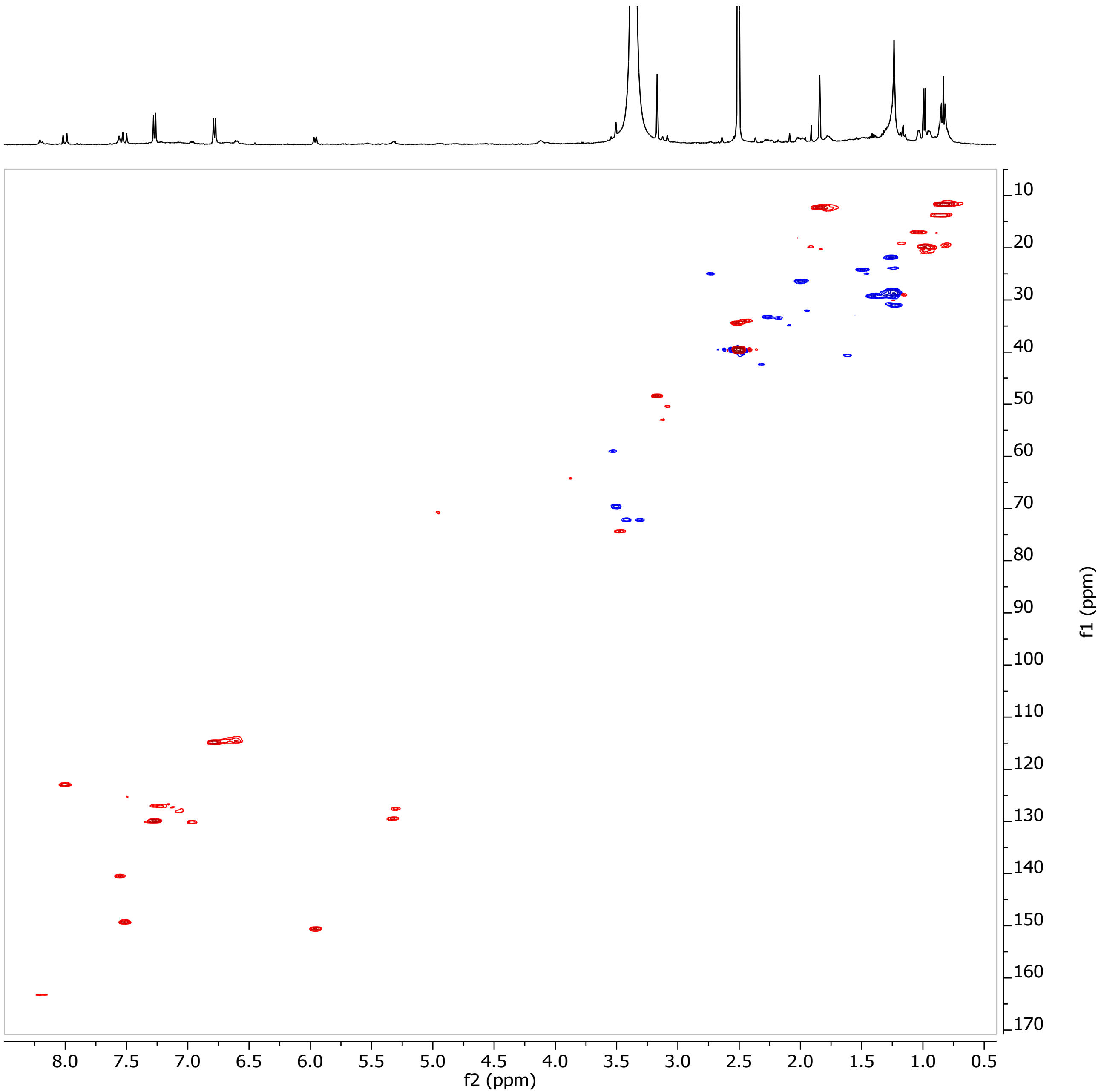

Figure S21. HSQC spectrum of **3** in DMSO-d_6 at 500 MHz.

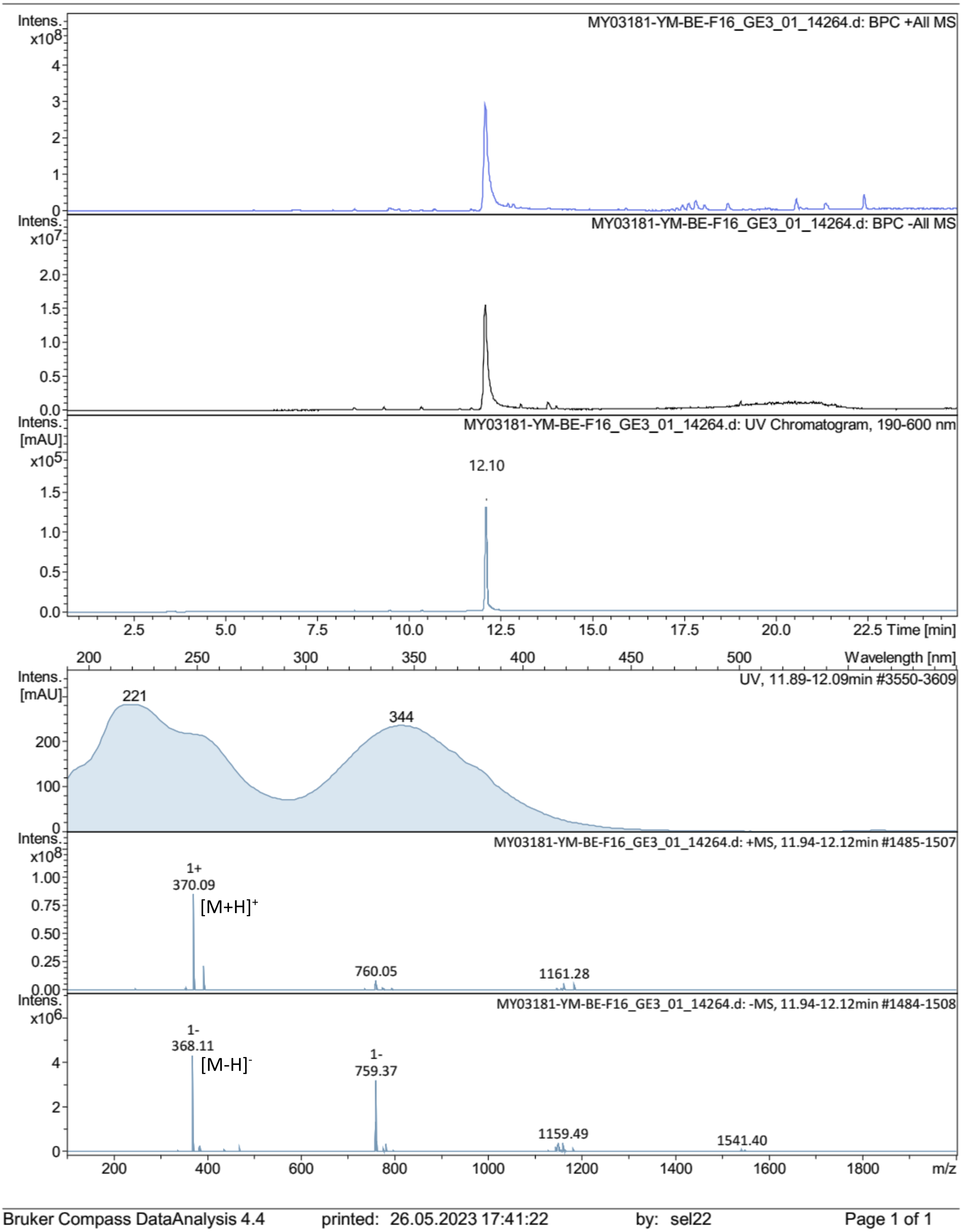

Figure S22. LRESIMS of **4**.

Generic Display Report

Analysis Info

		Acquisition Date	28.05.2023 10:03:17
Analysis Name	S:\DATA\MaXis\rto20_Rita Toshe\23_05\23_05_27\MY03181-YM-BE-F16_95_01_11718.d		
Method	pos_säure_10000_screening_ms_100_2500_line.m	Operator	ate06
Sample Name	MY03181-YM-BE-F16	Instrument	maXis
Comment	Screening01<br>Waters Acquity UPLC BEH C_18 1,7um 2.1x50mm		

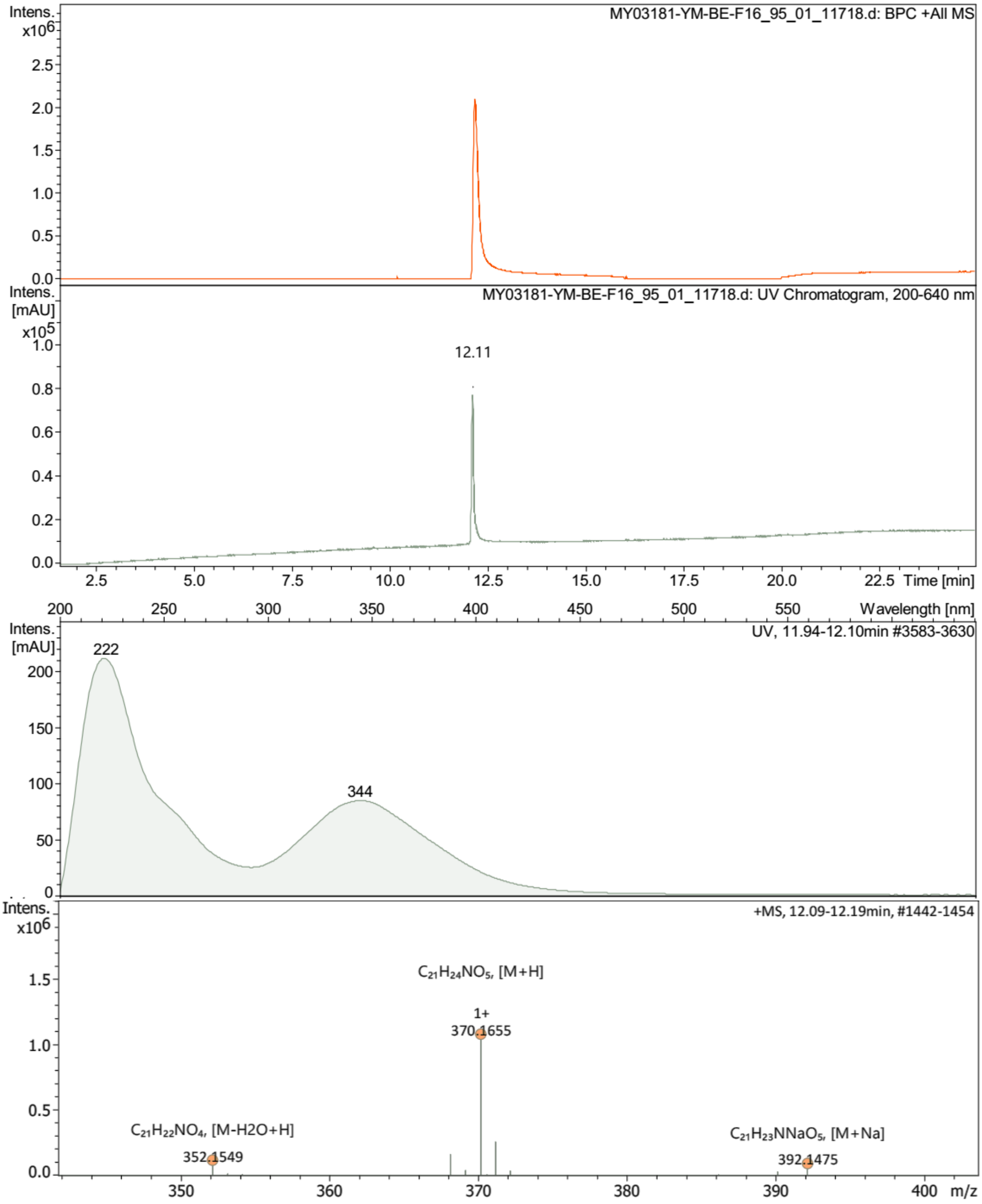

Bruker Compass DataAnalysis 6.1 printed: 30.10.2023 09:58:19 by: sel22 Page 1 of 1

Figure S23. HRESIMS of **4**.

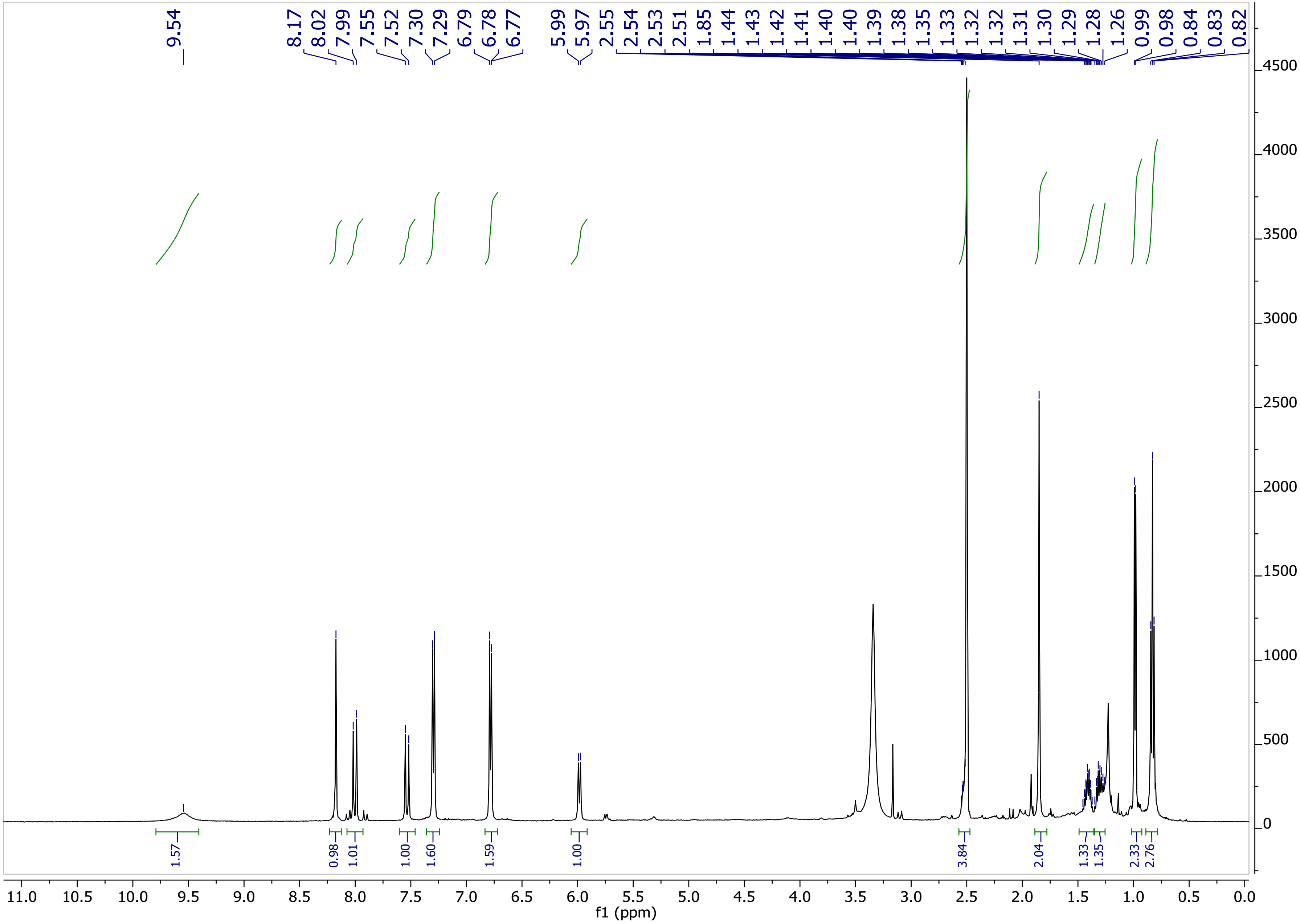

Figure S24. ^{1}H NMR spectrum of **4** in DMSO-d_6 at 500 MHz.

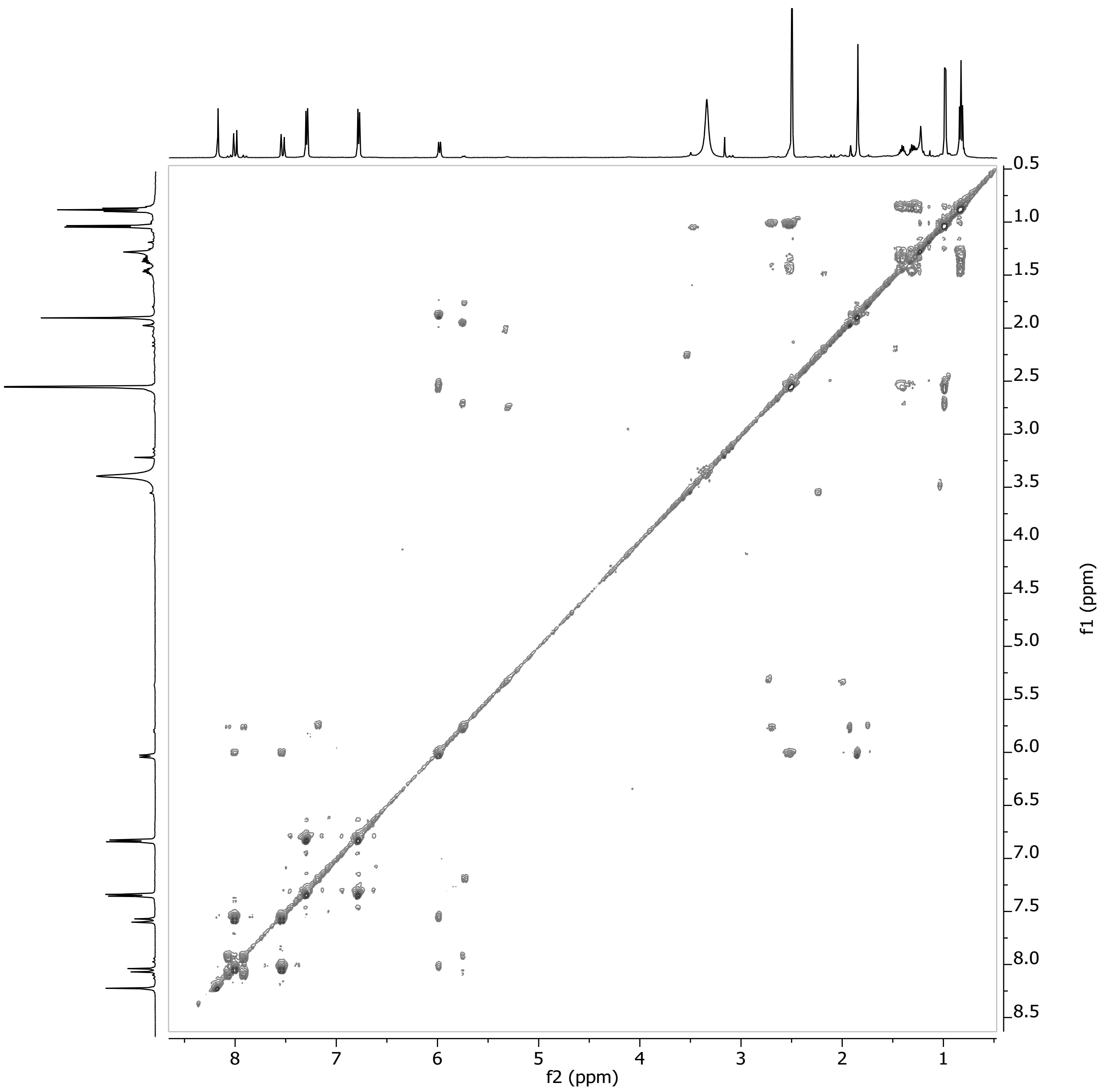

Figure S25. ^1H-^1H COSY spectrum of **4** in DMSO-d_6 at 500 MHz.

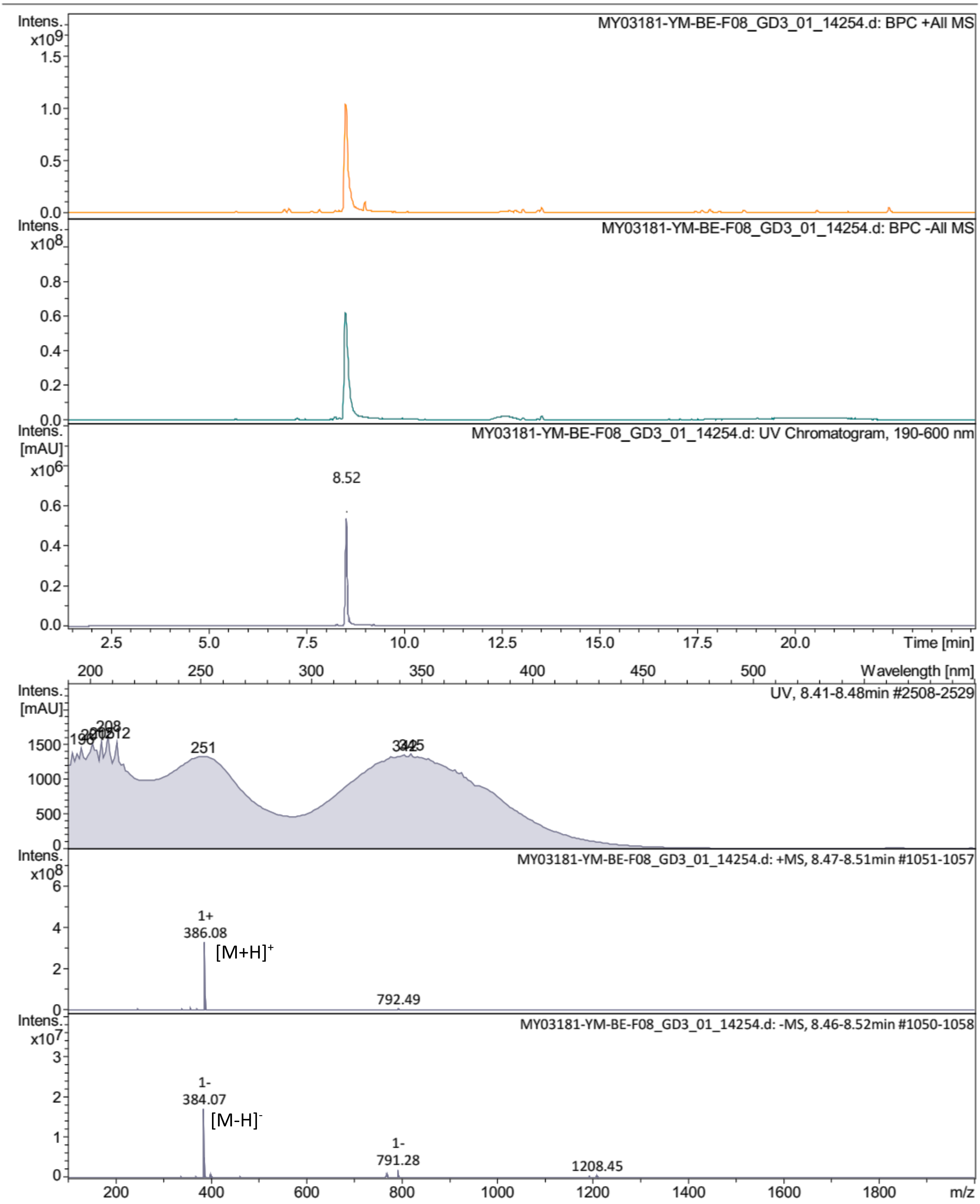

Bruker Compass DataAnalysis 4.4 printed: 26.05.2023 17:25:02 by: sel22 Page 1 of 1

Figure S26. LRESIMS of **5**.

Generic Display Report

Analysis Info

Analysis Name	S:\DATA\MaXis\rto20_Rita Toshe\23_05\23_05_27\MY03181-YM-BE-F08_87_01_11710.d	Acquisition Date	28.05.2023 05:55:37
Method	pos_säure_10000_screening_ms_100_2500_line.m	Operator	ate06
Sample Name	MY03181-YM-BE-F08	Instrument	maXis
Comment	Screening01<br>Waters Acquity UPLC BEH C_18 1,7um 2.1x50mm		

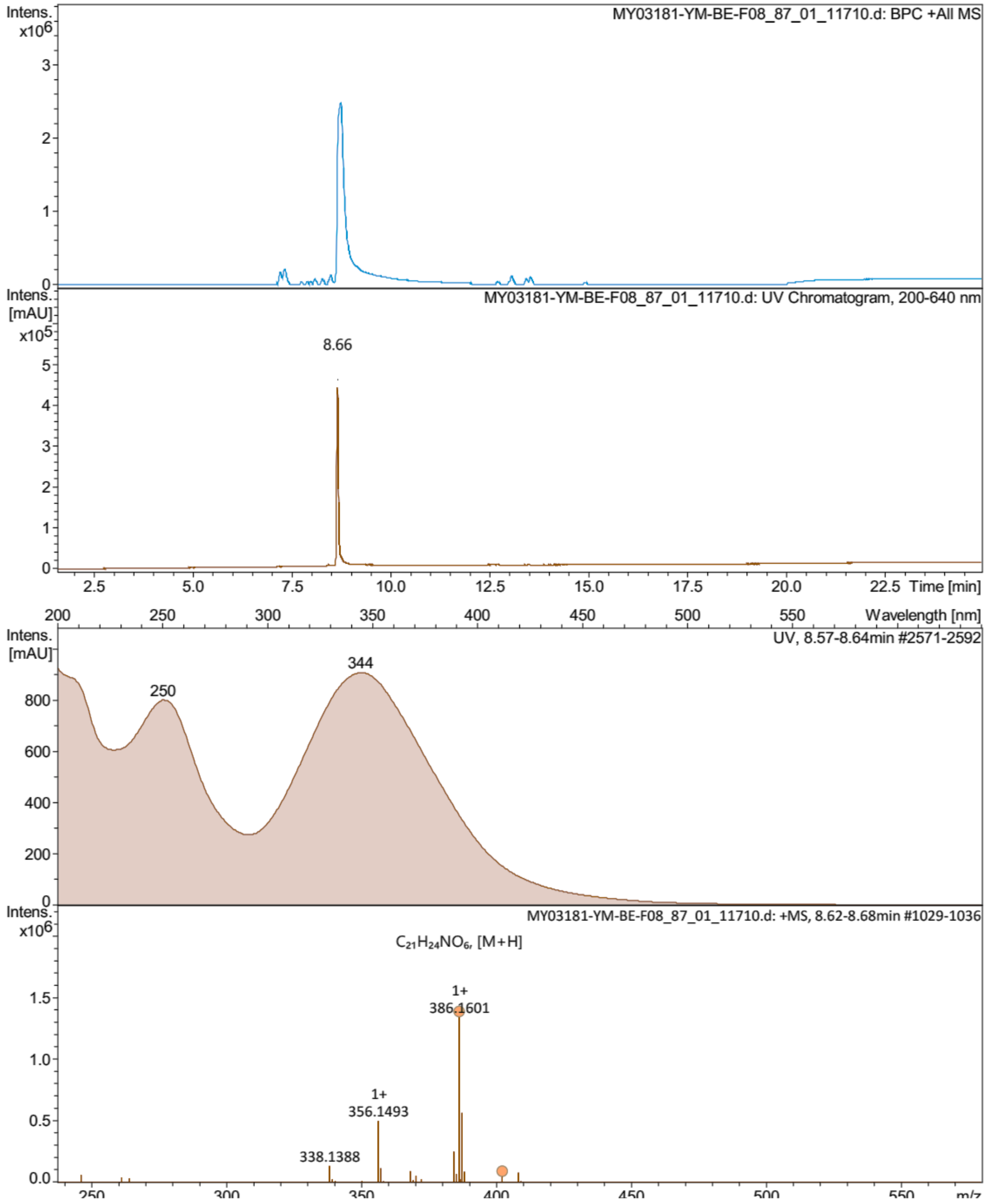

Bruker Compass DataAnalysis 4.4 printed: 30.05.2023 15:14:40 by: sel22 Page 1 of 1

Figure S27. HRESIMS of **5**.

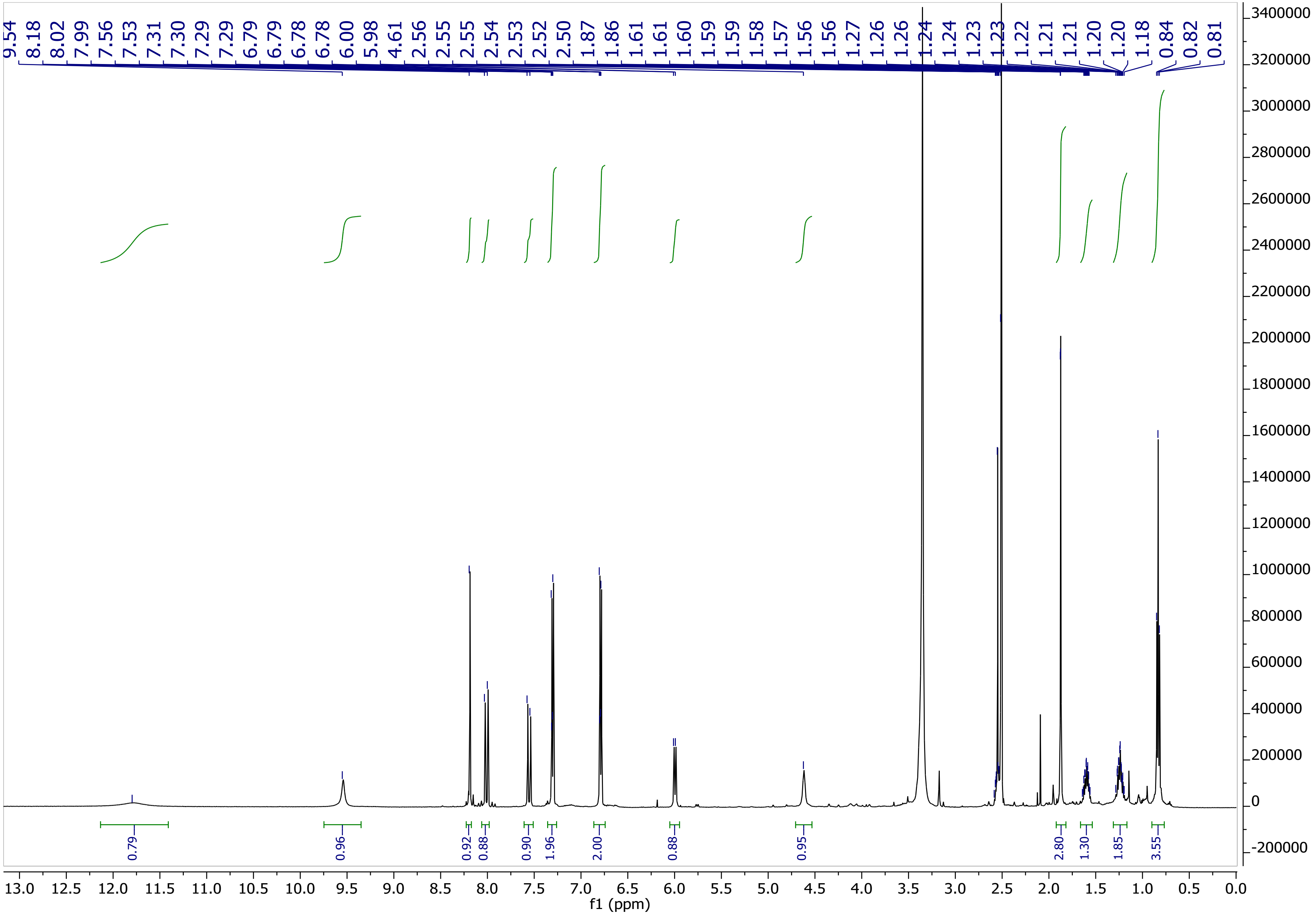

Figure S28. ^{1}H NMR spectrum of **5** in DMSO-d_6 at 500 MHz.

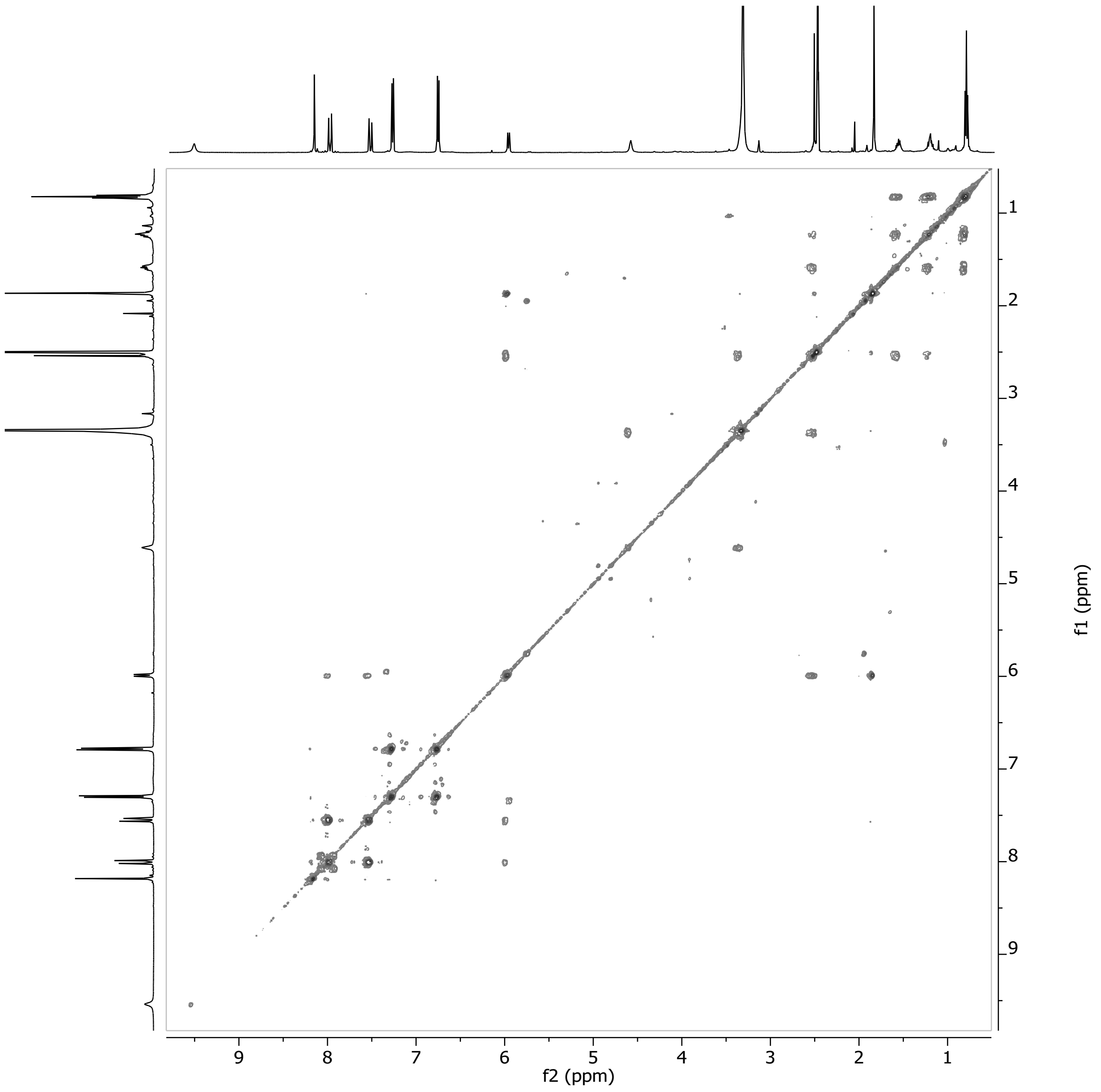

Figure S29. ^{1}H-^{1}H COSY spectrum of **5** in DMSO-d_6 at 500 MHz.

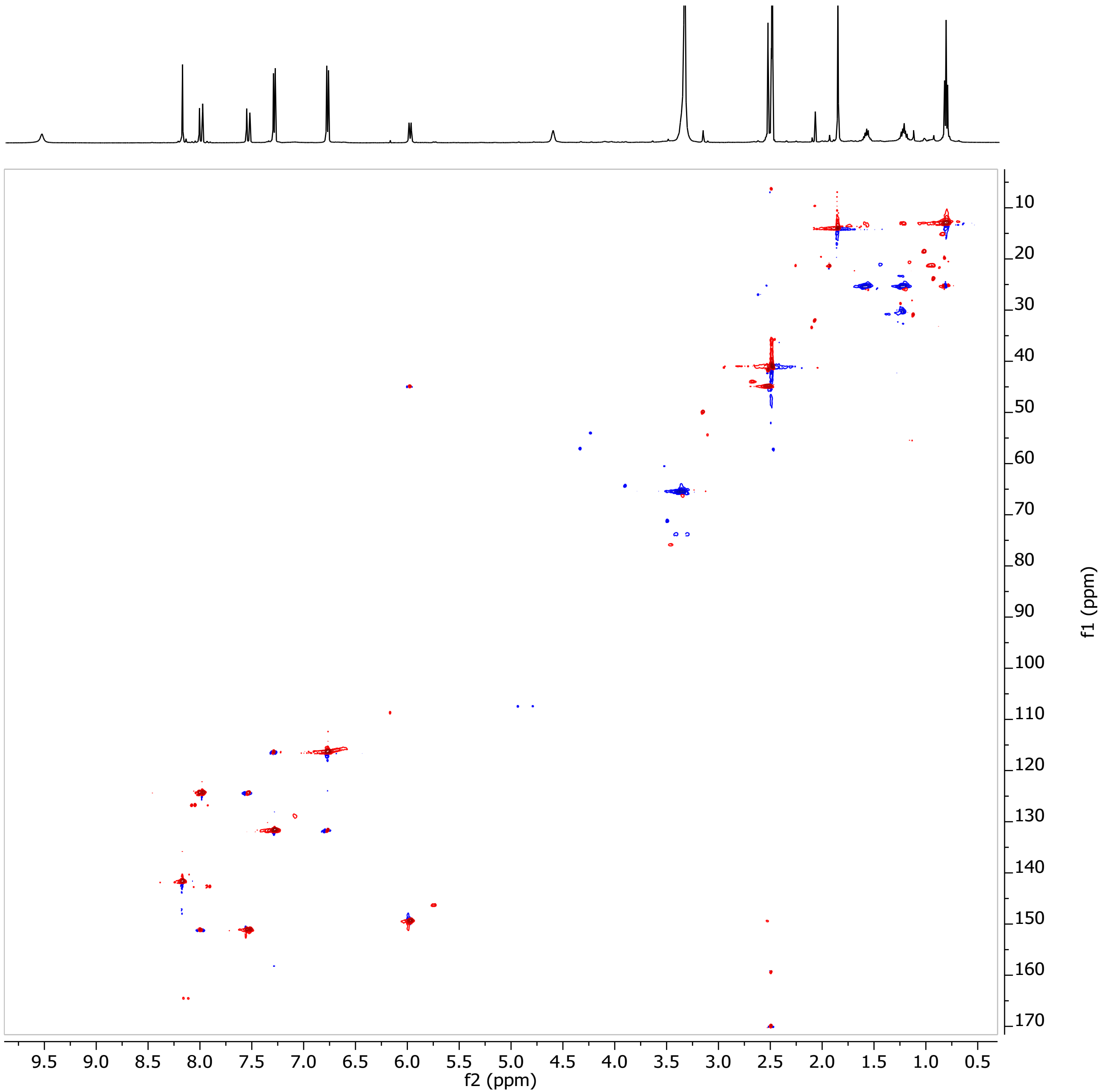

Figure S30. HSQC spectrum of **5** in DMSO-d_6 at 500 MHz.

Generic Display Report

Analysis Info

		Acquisition Date	10.05.2023 17:07:33
Analysis Name	S:\DATA\MaXis\rto20_Rita Toshe\23_05\MY03181-YM-CE-F13_19_01_11509.d		
Method	pos_säure_10000_screening_ms_100_2500_line.m	Operator	ate06
Sample Name	MY03181-YM-CE-F13	Instrument	maXis
Comment	Screening01<br>Waters Acquity UPLC BEH C_18 1,7um 2.1x50mm		

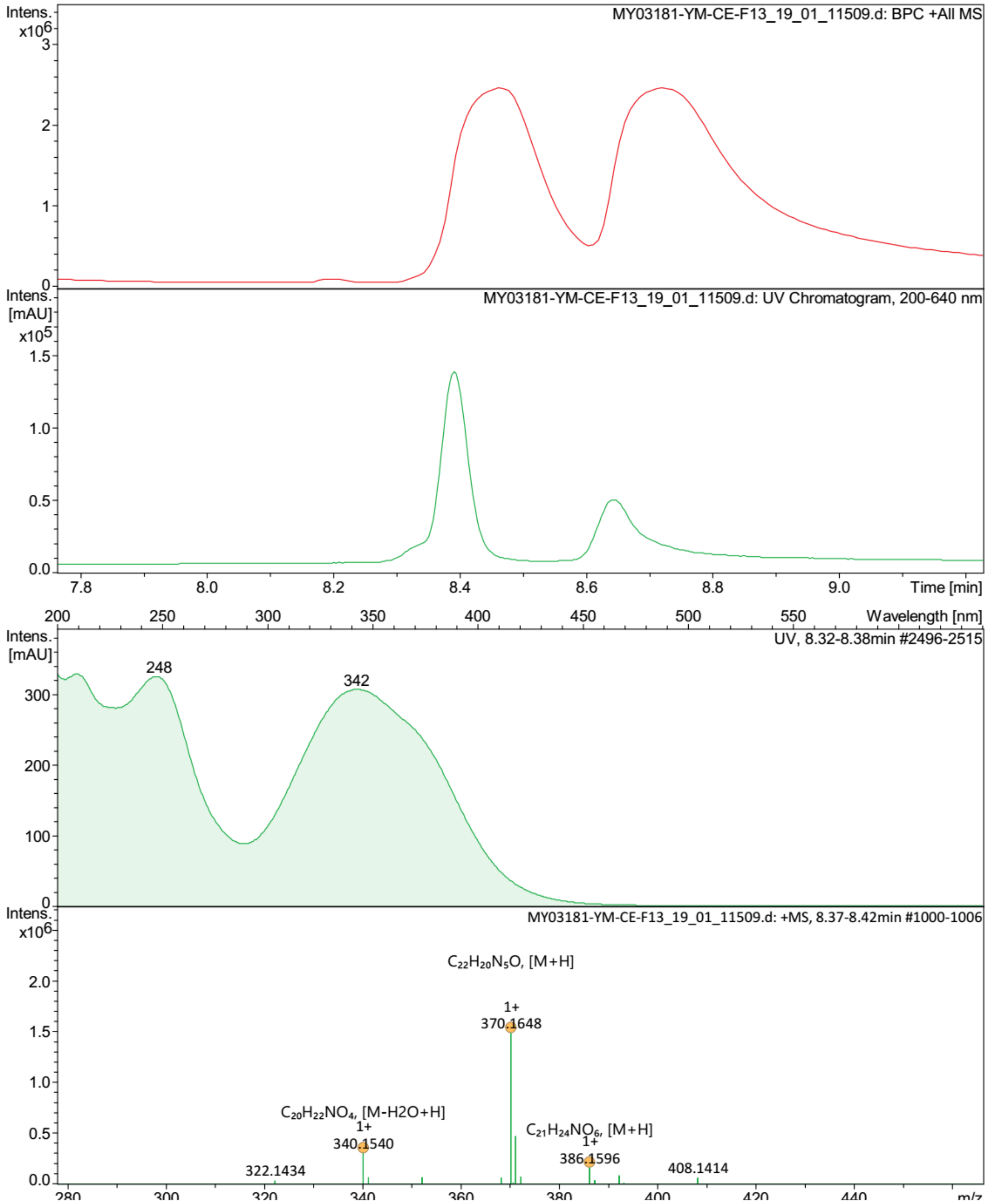

Figure S31. HRESIMS of **6**.

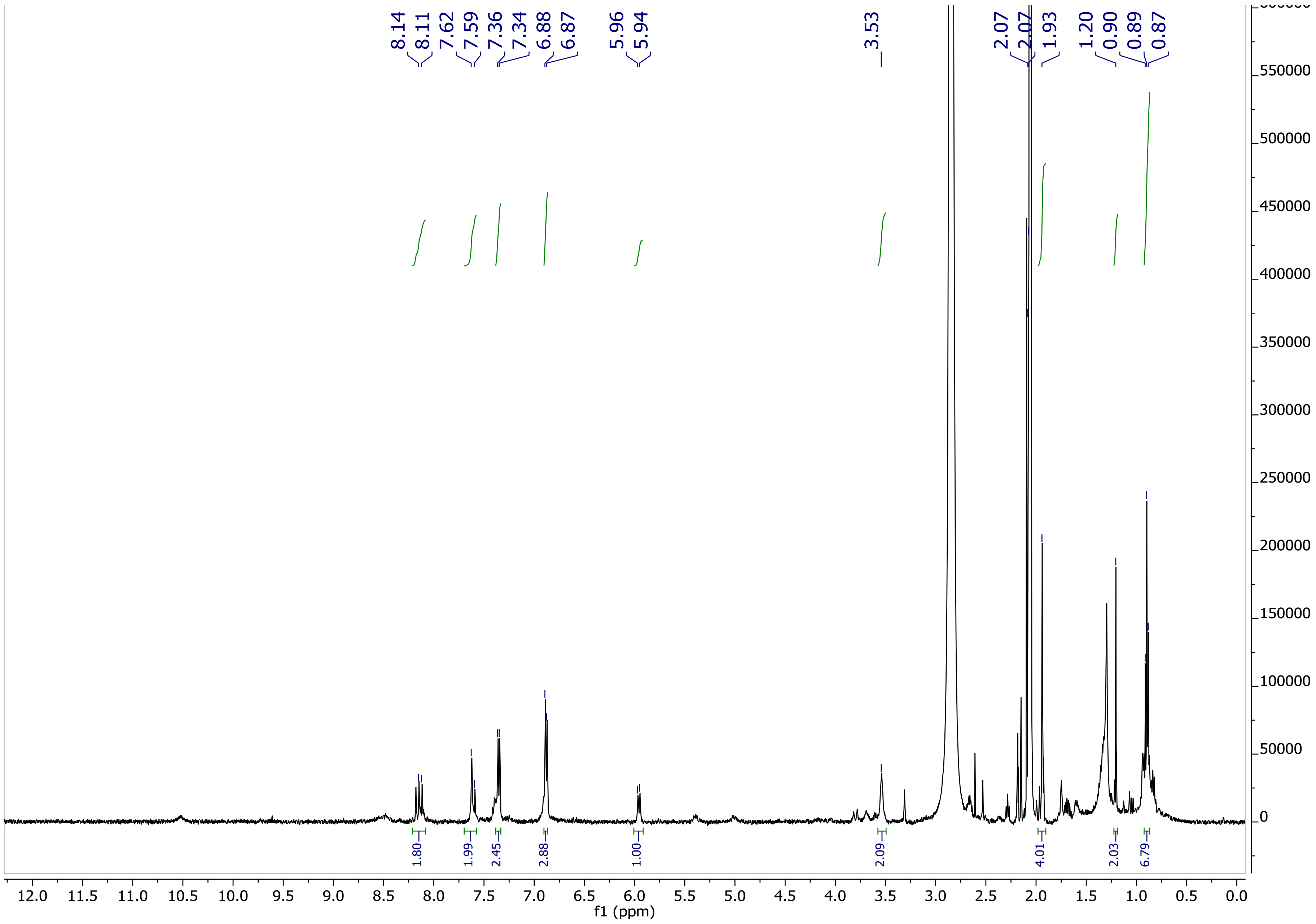

Figure S32. ^{1}H NMR spectrum of **6** in DMSO-d_6 at 500 MHz.

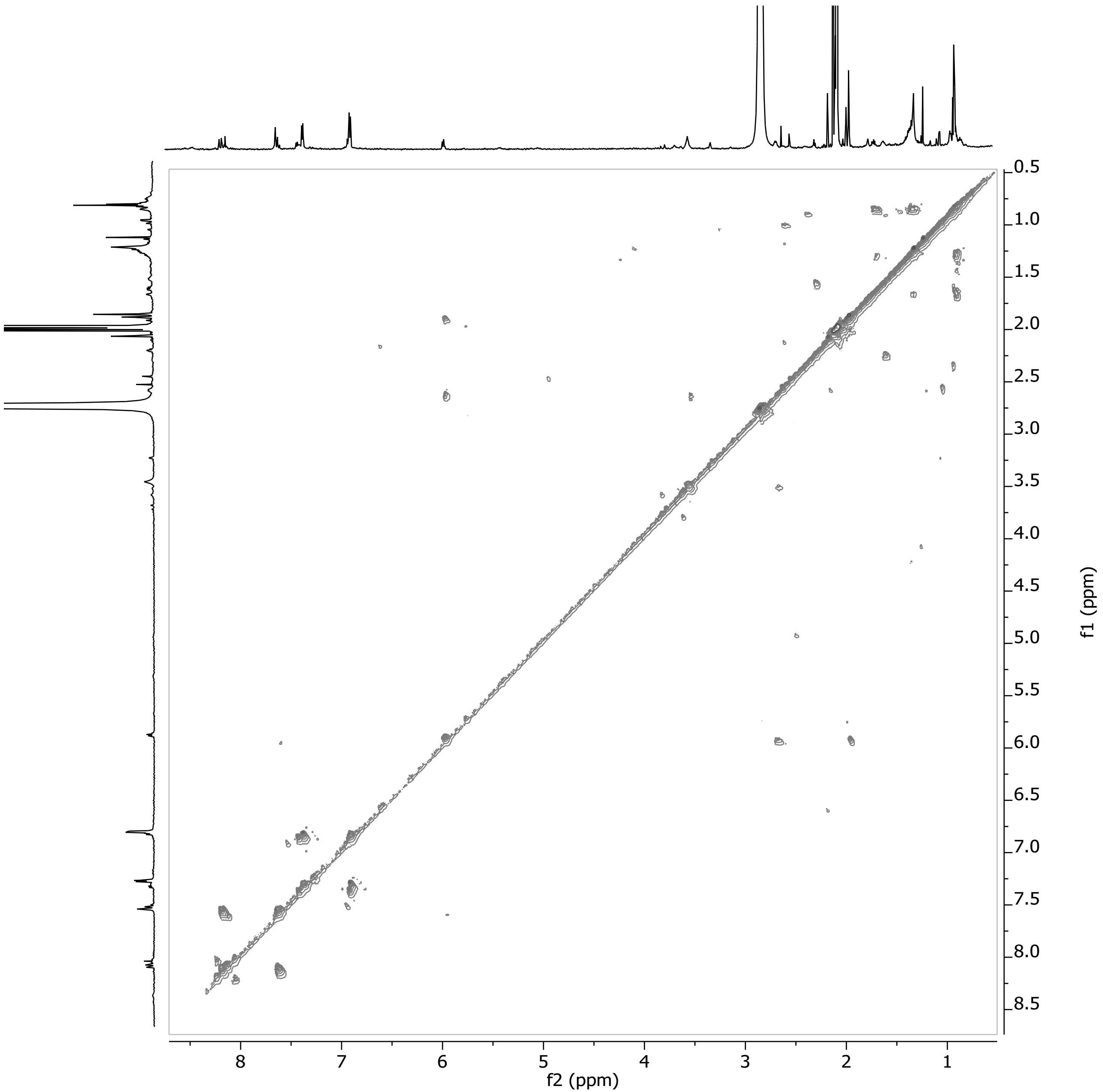

Figure S33. ^{1}H-^{1}H COSY spectrum of **6** in DMSO-d_6 at 500 MHz.

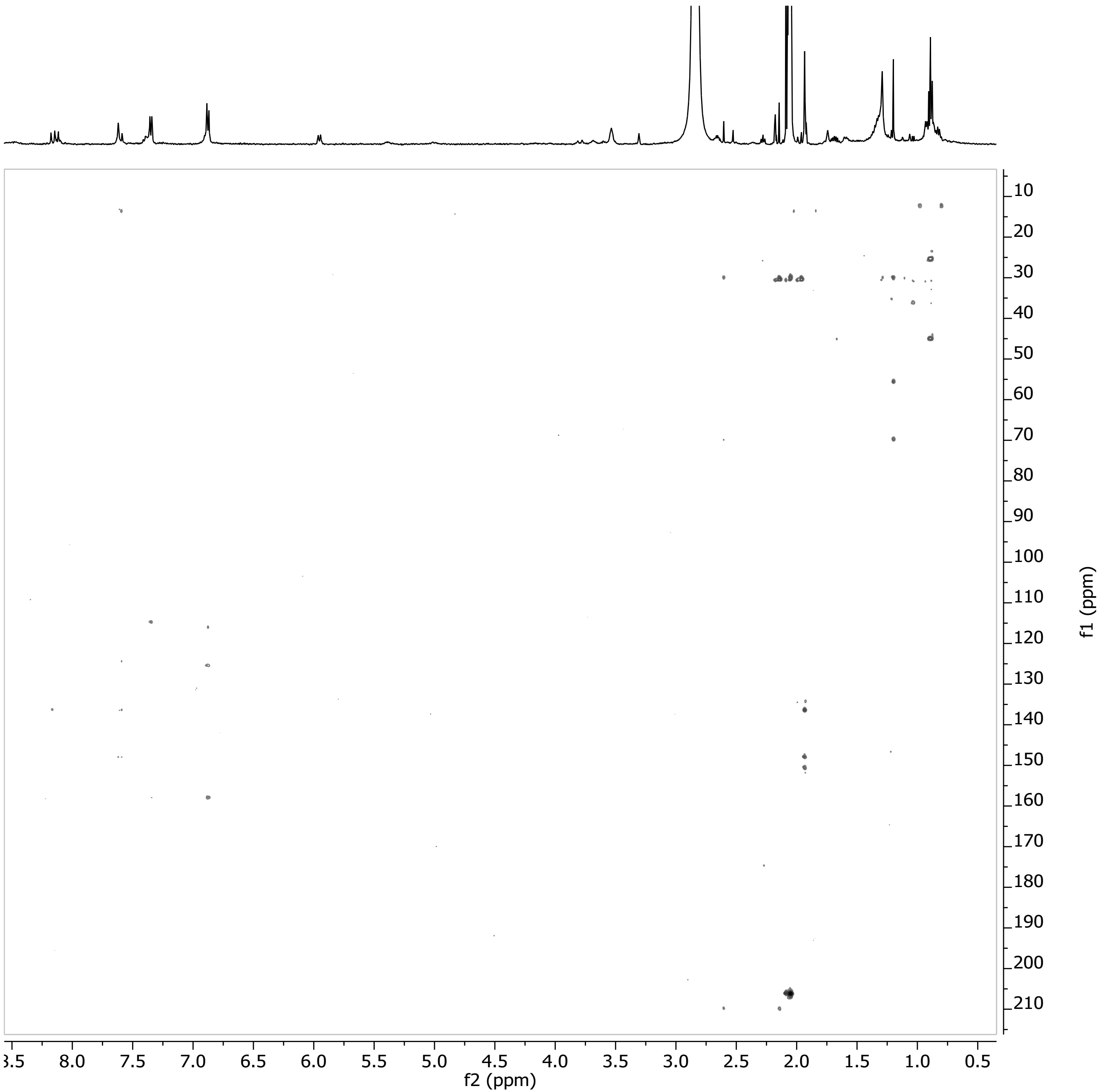

Figure S34. HMBC spectrum of **6** in DMSO-d_6 at 500 MHz.

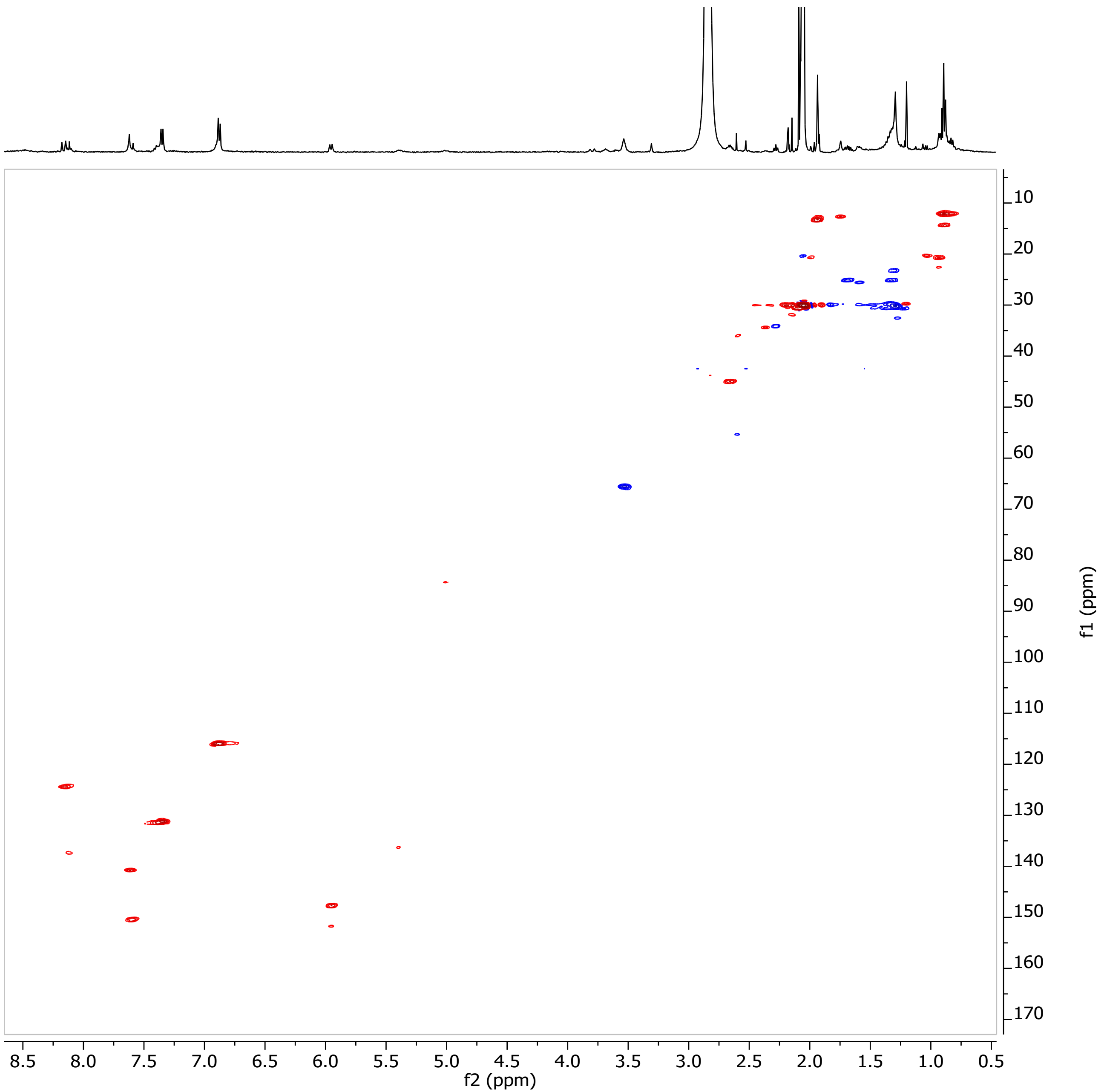

Figure S35. HSQC spectrum of **6** in DMSO-d_6 at 500 MHz.
